# Correlations in Somatic Hypermutation Between Sites in IGHV Genes Can Be Explained by Interactions Between AID and/or Polη Hotspots

**DOI:** 10.3389/fimmu.2020.618409

**Published:** 2021-02-02

**Authors:** Artem Krantsevich, Catherine Tang, Thomas MacCarthy

**Affiliations:** ^1^Department of Applied Mathematics and Statistics, Stony Brook University, Stony Brook, NY, United States; ^2^Laufer Center for Physical and Quantitative Biology, Stony Brook University, Stony Brook, NY, United States

**Keywords:** B cell receptor, activation-induced deaminase, computational immunology, immunoglobulin heavy chain, somatic hypermutation, overlapping hotspots

## Abstract

The somatic hypermutation (SHM) of Immunoglobulin (Ig) genes is a key process during antibody affinity maturation in B cells. The mutagenic enzyme activation induced deaminase (AID) is required for SHM and has a preference for WRC hotspots in DNA. Error-prone repair mechanisms acting downstream of AID introduce further mutations, including DNA polymerase eta (Polη), part of the non-canonical mismatch repair pathway (ncMMR), which preferentially generates mutations at WA hotspots. Previously proposed mechanistic models lead to a variety of predictions concerning interactions between hotspots, for example, how mutations in one hotspot will affect another hotspot. Using a large, high-quality, Ig repertoire sequencing dataset, we evaluated pairwise correlations between mutations site-by-site using an unbiased measure similar to mutual information which we termed “mutational association” (MA). Interactions are dominated by relatively strong correlations between nearby sites (short-range MAs), which can be almost entirely explained by interactions between overlapping hotspots for AID and/or Polη. We also found relatively weak dependencies between almost all sites throughout each gene (longer-range MAs), although these arise mostly as a statistical consequence of high pairwise mutation frequencies. The dominant short-range interactions are also highest within the most highly mutating IGHV sub-regions, such as the complementarity determining regions (CDRs), where there is a high hotspot density. Our results suggest that the hotspot preferences for AID and Polη have themselves evolved to allow for greater interactions between AID and/or Polη induced mutations.

## Introduction

The process of somatic hypermutation (SHM) is a key component of antibody affinity maturation in B cells. Activation induced deaminase (AID) initiates SHM by introducing C>U mutations in single-stranded DNA (ssDNA) at the antibody (Immunoglobulin or Ig) loci [reviewed in (1)]. These mutations are preferentially inserted at AID hotspots defined by the motif WRC (W = A/T, R = A/G), where the underline indicates the mutating nucleotide (2). Error-prone repair pathways including non-canonical Base Excision Repair (ncBER) and Mismatch Repair (ncMMR) act downstream of AID to introduce further mutations. In the absence of repair, the original C>U mutation results in a C>T transition (3). The ncBER pathway allows for C>G and C>A transversions at the original mutated site, whereas ncMMR can generate mutations at nearby A and T sites (4). Polymerase eta (Polη), which is part of the ncMMR pathway, introduces mutations preferentially at hotspots defined by the motif WA (5). Previous work has shown that where two AID hotspots are opposite each other on the two DNA strands, defined by the motif WGCW, mutations occur at a particularly high frequency (6). A greater abundance of WGCW motifs such as AGCT is observed in IgV subregions that mutate at high frequency, such as switch regions and the complementarity determining regions (CDRs) of the IGHV genes (7, 8). A recent study by ourselves found that co-localization of AID WGCW and Polη WA hotspots characterizes major differences between human IGHV germline genes (9).

Although it has been known for decades that SHM is strongly biased toward mutations in particular IgV subregions such as the CDRs (10), the more recent availability of high-throughput deep sequencing IgV data has enabled a more quantitative assessment of this bias (11). One approach has been to use nonproductive IgV sequences that have not undergone antigen driven selection, for example, by having nonsense-generating frameshifts in CDR3 that likely arose during V(D)J recombination (6). Mutational spectra from such nonproductive sequences have been shown to be surprisingly similar to those of productive sequences, as shown previously for human IGHV3-23 (12). Because the mutation spectra of nonproductive sequences should be unaffected by selection, these results suggest that intrinsic bias is dominant in SHM with selection playing at most a minor role.

There have been many previous efforts to characterize intrinsic SHM bias. Early attempts identified local contexts associated with higher mutation frequencies, such as the WRC and WA hotspot motifs (13, 14). Following the discovery of AID in 2000 (15), biochemical mutation assays demonstrated that the WRC motif, which had already been proposed as a hotspot, was in fact the preferred motif for AID (16). Subsequent identification of Polη as part of the downstream processing, led to characterization of the WA hotspot and its importance in IGHV genes (5). Recent analyses of deep sequencing data, in particular of silent (synonymous) sites and nonproductive V(D)J rearrangements, has led to systematic characterizations of mutability in SHM (11). For example, the S5F model (17) describes normalized mutability scores for all possible nucleotide changes in a context of +/−2 nt (there are 4^5^ = 1,024 such motifs of length 5). Interestingly, this approach found that these mutability scores were highly similar even between species as divergent as human and mouse (18).

However, there is another aspect of intrinsic bias that has received less attention to date, namely how mutations in one site affect mutability at other sites as SHM proceeds and as mutations accumulate in the IgV genes. Previous work has confirmed that an average of one mutation occurs per V gene per cell cycle in germinal centers (19). Presumably, single amino acid substitutions (as opposed to multiple substitutions) are more conservative at the structural level and thus less likely to cause detrimental changes. To achieve this, several mechanisms appear to have evolved to reduce mutability, including limited availability of the AID catalytic pocket (20) and tight regulation of AID activity to the early G1 phase of the cell cycle (21), among others.

Previously proposed mechanistic models of SHM suggest various ways in which mutations at one site may influence another site following an initial AID mutation. Mutations in one site may co-occur with mutations at another site more often (leading to a positive correlation) or less often (leading to a negative correlation) than would be expected by chance. For example, AID and nearby Polη hotspots might be expected to have a negative correlation, as follows. As described above, Polη mutations are entirely dependent on an initiating C>U mutation by AID (1). Replication bypass of the mutation will result in a C>T mutation. Alternatively, induction of ncMMR involves Exo1-mediated excision of a patch around the G-U mismatch. Subsequent recruitment of Polη to resynthesize the patch in an error-prone manner, leads to mutations preferentially in nearby WA hotspots. If the original G-U mismatch is then repaired, this would lead to a negative correlation between the site of the original mutation and WA hotspots within the excised patch, depending on whether replication bypass or ncMMR was induced. Other processes may lead to positive correlations, most obviously AID processivity, which has been demonstrated *in vitro* (16), and which may occur during *in vivo* transcription (22). Within any recurring patch of ssDNA, multiple C sites may also be deaminated due to processivity, leading to co-occurring, and therefore positively correlated, mutations.

Previous studies have evaluated correlations between sites using various measures. A common measure in genetic analysis is linkage disequilibrium (LD), defined as *D*(*X*;*Y*) = *p*(*x*,*y*) – *p*(*x*)*p*(*y*) for a pair of sites X and Y (23). Intuitively, if two nucleotides occur together (*p*(*x*,*y*)) more often than expected by chance (*p*(*x*)*p*(*y*)), as would be expected if they are in genetic linkage, then LD will be positive because *p*(*x*,*y*) > *p*(*x*)*p*(*y*). While the LD measure can distinguish the direction of the correlation, it cannot accurately detect its strength (see example in *Methods*). Another widely used metric is Mutual Information, defined as the amount of information obtained about one random variable through observing the other random variable. This metric captures the magnitude of the correlation even when the probabilities are small, but does not describe directionality.

For a metric that captures both direction and strength we defined a metric similar to mutual information that we call “mutational association” (MA). Using this metric, we identified two qualitatively different types of MA: relatively strong “short-range” MAs at distances 1–2 nt, and weaker “longer-range” MAs. Simulations showed that longer-range MAs arise primarily as a consequence of individual site mutabilities without explicit interactions between sites, in contrast to short-range MAs. We hypothesized that short-range MAs arose from mutations changing the mutability of neighboring sites. We verified this hypothesis using another simulation approach that considered step-by-step changes in mutability following each mutation. An extremely simple mechanistic model defined in terms of overlaps between adjacent AID and/or Polη hotspots largely predicts observed short-range MAs in human IGHV data. Such hotspot overlaps are most common in highly mutating IGHV subregions such as the CDRs, suggesting these are a major determinant of intrinsic mutational bias during SHM.

## Methods

### Mutational Aassociation

The standard measure of Mutual Information (MI) between two distributions X and Y is given by the formula

$$I(X;Y)=\underset{x,y}{\Sigma }p(x,y)log\left(\frac{p(x,y)}{p(x)p(y)}\right),$$

where the summation is performed over all *x* ∈ *X* and *y* ∈ *Y*.

In the case of mutual information between two mutation sites, for each of them we have only two possible events: in a given sequence nucleotide *X* either did mutate *(x = 1)* or it did not *(x = 0)*. Then we can expand the formula in the following way:

$$I(X;Y)=p(x=1,y=1)log\left(\frac{p(x=1,y=1)}{p(x=1)p(y=1)}\right)+p(x=1,y=0)log\left(\frac{p(x=1,y=0)}{p(x=1)p(y=0)}\right)+p(x=0,y=1)log\left(\frac{p(x=0,y=1)}{p(x=0)p(y=1)}\right)+p(x=0,y=0)log\left(\frac{p(x=0,y=0)}{p(x=0)p(y=0)}\right)$$

Now consider the first term of this formula (simplifying the notation to use *p(x,y)* instead of *p*(*x* = 1, *y* = 1), *p(x)* instead of *p*(*x* = 1) and *p(y)* instead of *p*(*y* = 1):

$$I^{1}(X;Y)=p(x=1,y=1)log\left(\frac{p(x=1,y=1)}{p(x=1)p(y=1)}\right)=p(x,y)log\left(\frac{p(x,y)}{p(x)p(y)}\right)$$

$log\left(\frac{p(x,y)}{p(x)p(y)}\right)$ is equal to zero if mutations at sites X and Y are independent (*p*(*x*,*y*) = (*p*(*x*)*p*(*y*))), greater than zero if mutations of both sites within the same sequence are observed more often than expected by chance (*p*(*x*,*y*) > (*p*(*x*)*p*(*y*))) and less than zero if mutations of both sites are observed together less often than expected by chance (*p*(*x*,*y*) < (*p*(*x*)*p*(*y*)). As a result, the $log\left(\frac{p(x,y)}{p(x)p(y)}\right)$ term of *I*^1^ can be considered as a correlation-like measure, though it is sensitive to noise if the probabilities involved are very small. It is balanced out by the *p*(*x*,*y*) term, which can be considered as a measure of the strength of the observed correlation. One can notice that the term $log\left(\frac{p(x,y)}{p(x)p(y)}\right)$ functions similarly to the linkage disequilibrium metric *D*(*X;Y*) = *p*(*x,y*) – *p*(*x*)*p*(*y*), though unlike LD, $log\left(\frac{p(x,y)}{p(x)p(y)}\right)$ measures the relative, rather than absolute, amplitude of the change.

However, a weakness of both *I*^1^ and LD formulas is that they are positively biased. Consider a hypothetical pair of nucleotides X and Y with corresponding *p*(*x*) = *a* and *p*(*y*) = *b*. If they mutated completely independently from each other, the likelihood of both mutations appearing simultaneously would be *p*(*x*,*y*) = *ab*. Now consider two scenarios: mutations X and Y occur simultaneously

*n* times less often than expected (*p*^-^(*x*,*y*) = *ab*/*n*) and
*n* times more often than expected (*p*^+^(*x*,*y*) = *abn*),

(*n* > 1). Now we have

$$I^{1}(X;Y)=\frac{ab}{n}*log\left(\frac{ab}{n*ab}\right)=\frac{ab}{n}*log\left(\frac{1}{n}\right)=-\frac{ab}{n}*log(n)$$

$$I^{1}(X;Y)=abn*log\left(\frac{abn}{ab}\right)=abn*log(n)=-I^{1}(X;Y)*n^{2}$$

$$D^{-}(X;Y)=\frac{ab}{n}-ab$$

$$D^{+}(X;Y)=abn-ab=n(ab-\frac{ab}{n})=-D^{-}(X;Y)*n$$

Despite the fact that both positive and negative deviations had the same scale, the positive signal is *n*^2^ times stronger than the negative one for *I*^1^ and *n* times stronger for LD. This leads to a positive bias for both metrics. In order to avoid this bias, we replace the “strength” term *p*(*x*,*y*) in *I*^1^ with *p*(*x*)*p*(*y*) and this is our “mutational association” (MA):

$$MA(X;Y)=p(x)p(y)log\left(\frac{p(x,y)}{p(x)p(y)}\right)$$

By *MA_g_*(*X*;*Y*), we refer to the mutational association between nucleotides at the positions *X* and *Y* in the gene *g*.

### Generation of Independent Simulated Data Set

We aim to generate a simulated dataset in which mutations are independent from each other. The simulated dataset should also match the original data for each gene in the following: 1) total number of sequences *S*; 2) number of sequences *S_i_* containing exactly *i* mutations for any *i*; 3) observed mutation frequency at each position. In our approach we maintain (1) and (2) exactly, while (3) is approximated. Assuming that we have a set of genes *G* and for each gene g ∈ *G* we have the corresponding vector of mutability scores *MutScores_g_* (defined below), the total number of sequences *S_g_*, the maximal number of mutation per sequence *M_g_* and for any *i*, the number of sequences containing exactly *i* mutations *S_g,i_*

$$(\overset{M_{g}}{\underset{i}{\Sigma }}S_{g,i}=S_{g}).$$

In order to generate an Independent dataset:

1. Define the probability distribution *P_g_* as a normalized vector *MutScores_g_*
2. Randomly draw *i* mutations without replacement according to *P_g_*.
3. Save the resulting vector of mutations.
4. Repeat 2 and 3 until we have *S_i_* vectors.
5. Repeat 2–4 for all *i*.
6. Repeat 1–5 for all g ∈ *G*.

Our original dataset provides all the required parameters except for the vectors *MutScores_g_*. In the first instance we use the observed mutation frequencies for each gene as an initial approximation of *MutScores_g_*. Because drawing mutations without replacement changes the distribution for the subsequent remaining mutations, this results in a discrepancy between the original observed mutation frequencies and those of the simulation output (**Figure S4**). However, we noted that the relationship between these two mutation frequencies could be well approximated using a cubic polynomial, suggesting we could adjust *MutScores_g_* for each gene using standard cubic regression *y* = *a*_3_*x*^3^ + *a*_2_*x*^2^ + *a*_1_*x*^1^ + *a*_0_ as shown in **Figure S4**. Using this adjustment, we decreased average RMSD between original and Independent mutation frequencies from 0.0138 to 0.0026.

Now the full simulation can be summarizsed as follows:

- For a given gene g define *MutScores_g_* = *MutationFrequency_g_*
- Perform steps 1–5.
- Using output of step II, use cubic regression to obtain new vector *MutScores_g_*
- Using new vector *MutScores_g_* repeat steps 1–5
- Repeat steps I–IV for all genes.

### Hotspot Confirmation

In order to evaluate our hypothesis regarding the influence of the DNA sequence context on certain mutational association patterns, we used the following approach. Assume there is a pair of nucleotides (*i* and *j*) and we have a hypothesis for the local context *C* leading to a higher or lower MA. We consider all appearances of *i* and *j* at distance *k* (distance at which they are placed apart in *C*), and separate these into two sets: those with context C (*ContextMA_g_* (*i,j,k,C*)) and those without (*NonContextMA_g_* (*i,j,k,C*)). We compare the context and non-context sets using a difference of means (Mann–Whitney U) test, accepting the hypothesis if the two distributions are significantly different and the context of one is shifted in the predicted direction. This approach was used in column 9 of **Table 2**.

An alternative approach is to compare *ContextMA_g_* (*i,j,k,C*) sets for the original and Independent data using a paired Wilcoxon signed-rank test to test whether the original data is significantly shifted in the predicted direction. This approach was used in column 5 of **Table 2** and column 9 of **Table 3**.

### Distribution of Overlapping Hotspots Within IGHV Genes’ Sequences

In order to analyze localization of generalized overlapping hotspots (**Figure 8**), we began by using the method described in (9), which was used there for WGCW and WA motifs. In this method, a moving window of size 31nt (i.e., a middle site +/- 15nt) is used throughout a gene, and for each position the number of the occurrences of each overlapping hotspot (OHS) motif (see **Table 1**) within the window is counted. Then, a weighted sum of these values is calculated, and divided by the window size. In other words, each sequence is represented as an OHS distribution profile where each value measures the hotspot density in the neighborhood around each position in the sequence. The weights are proportional to the expected abundance of each motif under the assumption that each nucleotide is equally likely. For example, we would expect on average 1 in 16 dinucleotides NN to be TA (14×14), while a 4-mer NNNN has only a 1/128 chance to be a WACC motif. Using this reasoning we split all OHS into 4 groups: 1) the least abundant motifs (WACC, GGTW, WRCRC, and GYGYW) were assigned with weight equal 1; 2) WGCW, WARC, GYTW, TAC, GTA, WRCA, TGYW were assigned with weight equal 0.5; 3) WAC, GTW, WAA, and TTW have weights equal to 0.25 and 4) the most abundant motif TA has weight equal to 0.125.

**Table 1:** Enumeration of all possible AID and/or Polη overlapping hotspots.

		W	R	C		One-sided	Mutual	Sequential			G	Y	W			One-sided	Mutual	Sequential
	W	A				WARC	X	X					T	W		GYTW	X	X
		W	A			WAC	X	X				T	W			GTW	X	X
		T	W			TAC	X	X				W	A			GTA	X	X
			T	W		X	X	X			W	A				X	X	X
W	R	C				X	X	WRCRC					G	Y	W	X	X	GYGYW
	W	R	C			X	X	WACC				G	Y	W		X	X	GGTW
		G	Y	W		X	X	X			W	R	C			X	X	X
			G	Y	W	X	WGCW	X		W	R	C				X	WGCW	X
		**W**	**A**			**One-sided**	**Mutual**	**Sequential**				**T**	**W**			**One-sided**	**Mutual**	**Sequential**
	W	A				WAA	X	X					T	W		TTW	X	X
		T	W			X	TA	X				W	A			X	TA	X
W	R	C				X	X	WRCA					G	Y	W	X	X	TGYW
		G	Y	W		X	X	X			W	R	C			X	X	X

There are eight theoretically possible overlaps for the WRC/GYW motif. Because AID hotspot has 2 context nucleotides, another Polη or AID hotspot could potentially overlap it in two ways. Since WA/TW has only one context nucleotide, there are only four theoretically possible overlaps. Conflicts between a candidate for an overlap and WRC/GYW or WA/TW are highlighted in red. Conflicts in the mutating nucleotide may either result in sequential overlap or make the overlapping hotspot structure impossible.

To ensure that the distribution profiles were of equal length (in order to make aggregation of data for multiple genes possible), we used the standard gapped alignments with unique codon numbering from IMGT and linear interpolation, a curve-fitting method, to adjust for differences in IGHV sequence lengths using the R function *approx*. Lastly, allelic variants were ignored and only *01 alleles were used.

### Sequences Used for Analysis

Since analysis of pairwise interactions between mutation sites requires a relatively large amount of data, we used a cutoff of at least 5,000 productive sequences per allele for the analysis. This resulted in a dataset with 31 alleles (5 from family 1, 14 from family 3, 10 from family 4, 2 from family 5) and containing a total of 399,505 sequences with 65,071 as the maximum (IGHV3-23*01) and 5,239 as the minimum (IGHV3-53*01).

## Results

### Definition of Mutational Association (MA)

We defined our correlation measure, “mutational association” (MA), as follows. For a pair of mutation sites X and Y,

$$MA(X;Y)=p(x)p(y)log\left(\frac{p(x,y)}{p(x)p(y)}\right),$$

where *p*(*x*) is an observed probability of the site X mutating *p*(*y*), is an observed probability of the site Y mutating and *p*(*x,y*) is an observed probability of sites X and Y both mutating within the same sequence.

MA has two parts that represent different characteristics: correlation ($log\left(\frac{p(x,y)}{p(x)p(y)}\right)$) and strength (*p*(*x*)*p*(*y*)). Choosing the strength term to be *p*(*x*)*p*(*y*) rather than *p*(*x,y*) as in mutual information, eliminates an implicit bias (see *Methods*).

### MA Values Are Highest for Pairs of Sites at Short Distances

**Figure 1:**
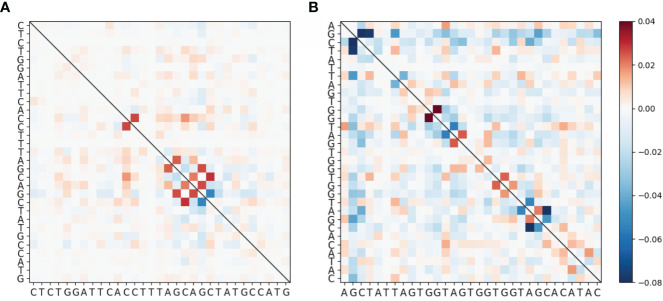
Heatmap showing MA for the IGHV3-23*01 allele around **(A)** CDR1 and **(B)** CDR2. These data have been corrected by subtracting the MA values from the “Independent” simulated data set (see *Materials and Methods*) from the MA values of the original data. The full gene heatmaps for original uncorrected MA values, as well as for corrected MA values and MAs for simulated datasets, are shown in **Figures S1 **and** S2**.

**Figure 2:**
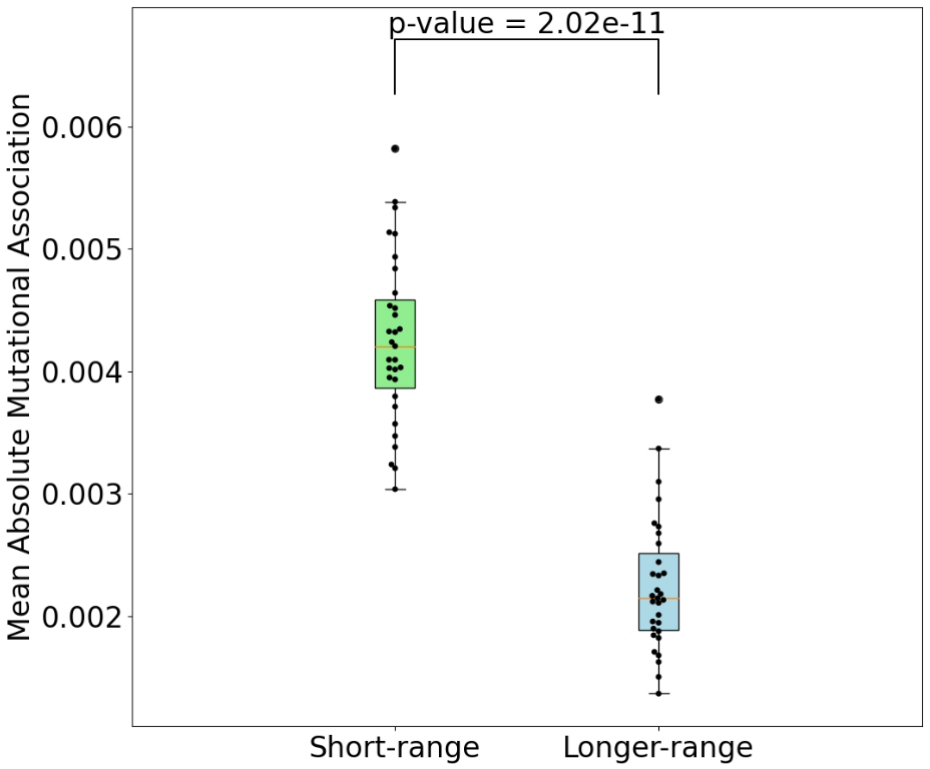
Comparison of mean absolute MA for short-range interactions (1-2 nt between nucleotides) and all others. P-value shown is a result of Mann-Whitney (difference of means) test.

To evaluate mutational association (MA) between sites in IGHV genes, we used a previously published high quality human Ig Repertoire sequencing dataset (9). The sequences analyzed here were sequenced as described in Vergani et al. (24) and preprocessed as described in Tang et al. (9), although here we use both productive and nonproductive IGHV sequences. Briefly, sequencing data originated from IGHV RNA samples tagged with Unique Molecular Identifiers (UMIs). For preprocessing, Immcantation framework packages were used for quality control, clonotype analysis and filtering out novel alleles, while IMGT/V-Quest was used for determining IGHV genes and CDR3 boundaries. Here we chose 31 IGHV alleles (across 26 genes) for which we had at least 5,000 (productive) sequences per gene. Because we found no qualitative difference between productive and nonproductive sequences with respect to MA by performing analysis for both sets separately, we only show results for the more abundant productive sequence data (see **Figure S6** for the nonproductive analysis summary). We calculated pairwise MA values for all sites in each IGHV gene. As an example, MA values for CDRs 1 and 2 of the IGHV3-23 gene are shown in **Figure 1A** (CDR1) and **Figure 1B** (CDR2). The values shown in **Figure 1** have been corrected to remove statistically expected correlations (extracted from the “Independent” simulations, as explained below) that arise from high pairwise mutation frequencies. Here we found that almost all of the high MA values correspond to sites that are within 1 to 2 nt of each other—these are the values close to the diagonal in **Figure 1**—whereas the values further from the diagonal tended to be relatively weak. **Figure 2** shows that indeed mutation sites placed within 1–2 nt of each other tend to have stronger MA signals across all IGHV genes analyzed.

**Figure 3:**
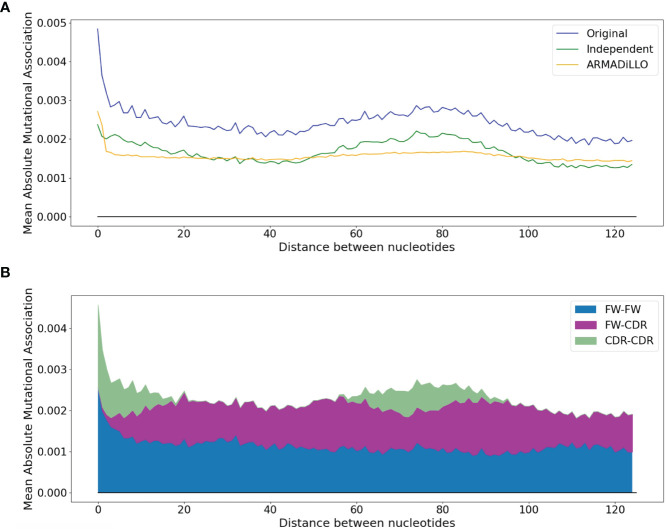
Dependence of the mean absolute MA on the distance between nucleotides. **(A)** Comparison between the original dataset and the two simulated datasets (ARMADiLLO and Independent). **(B)** Breakdown of contributions to the “original” line of **(A)** made separately by pairwise measures for FW-FW, FW-CDR, and CDR-CDR interactions. CDR-CDR interactions are aligned with the main peaks.

To illustrate this trend in greater detail, the blue curve in **Figure 3A** shows how the mean absolute MA value varies with the distance between sites. Here, there is a pronounced high value corresponding to distances of 1 to 2 nt, which then drops for more distant interactions. The MA values then rise again, reaching a lower peak at distances of ~75 nt. Because these more distant peaks are dominated by interactions between sites in the CDRs (**Figure 3B**), it suggests that at least part of the MA signal might be generated by pairs of sites that both had high mutation frequencies but which were not necessarily correlated, positively or negatively, beyond what would be expected. We next evaluated the independent effect of high mutation frequencies, as well as the distribution of mutations per sequence, which may also contribute to MA. We therefore performed simulations that, based on the observed site-by-site mutation frequencies for each IGHV gene, produce simulated datasets that have almost exactly the same mutation frequencies at each site and exactly the same distribution of mutations per sequence (see *Methods*). In the simulated data we should not observe any correlations between sites beyond what is expected, because the mutations are generated independently of one another. The resulting MA values are shown in the green curve of **Figure 3A**, labeled “Independent”. The simulated “Independent” curve follows the same pattern as the original data (blue curve), with the exception of sites within 1 to 2 nt of each other, where the original absolute MA values are much higher. The gap between the two (“Independent” vs original) curves is almost constant for all distances >2 nt, and can largely be explained as a consequence of sampling bias since the gap, measured as root mean squared difference or RMSD, becomes smaller with increased sample size (Spearman correlation, −0.61, p-value = 0.0003—see **Figure S3A**). In other words, if we had as much data for every allele as we have for IGHV3-23*01 (> 65K sequences), then the gap would become negligible (**Figure S3B**). We will hereafter refer to the above-expected correlation levels between nearby (1–2 nt) sites as “short-range MAs”, in contrast to “longer-range MAs” (> 2nt), which are not substantially different from what is expected in the case of Independent mutations. In order to correct the observed MAs for what is expected, we subtracted the Independent MA values from the original MA values, leading to “corrected” data that we used for the subsequent analysis.

### Short-Range MAs Are Associated With Interactions Between Overlapping Hotspots

**Figure 4:**
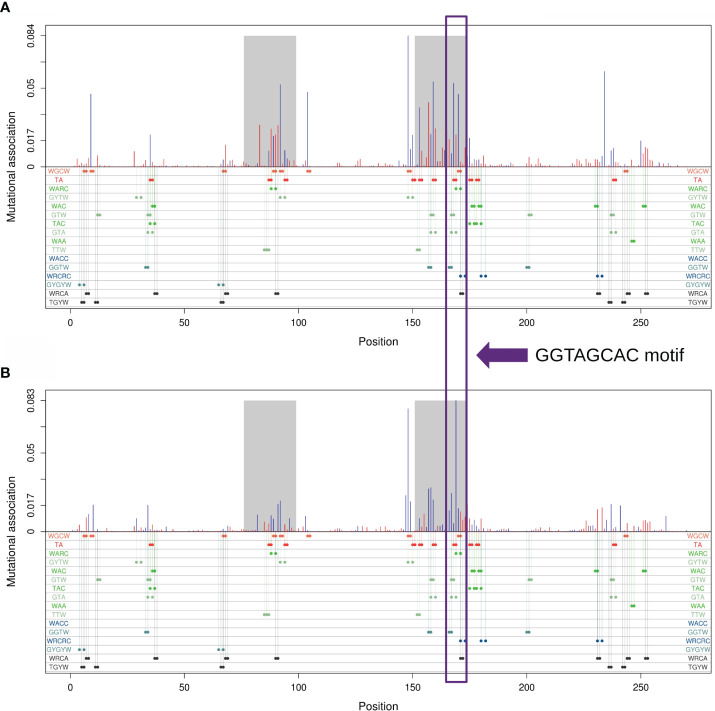
Mutational association (MA) for pairs of nucleotides within IGHV3-23*01. **(A)** MAs between adjacent nucleotides. **(B)** MAs between nucleotides separated by exactly one nucleotide. Red bars represent positive MAs, blue bars represent negative MAs, where each bar is aligned with the first nucleotide of the pair. CDR1 and CDR2 are highlighted in gray. Positions for all generalized overlapping hotspots are shown as colored dots below the x-axis.

We further sought to identify which subregion within each IGHV gene had the strongest MA effects. As an example, **Figure 4A** shows MA values for adjacent sites within the IGHV 3–23 × 01 gene, whereas **Figure 4B** shows for sites that are one nucleotide apart from one another. The results for IGHV3-23 suggested that the strongest MA values would be in the CDRs, although other smaller subregions, for example within FW3, also had some high values [this subregion of FW3 is known to have a high mutation frequency and is often referred to as “CDR4” (25)]. **Figure 4** also shows that the CDRs are enriched for various motifs involving overlapping hotspots for AID and/or Polη. We discuss these hotspot overlaps in greater detail below.

**Figure 5:**
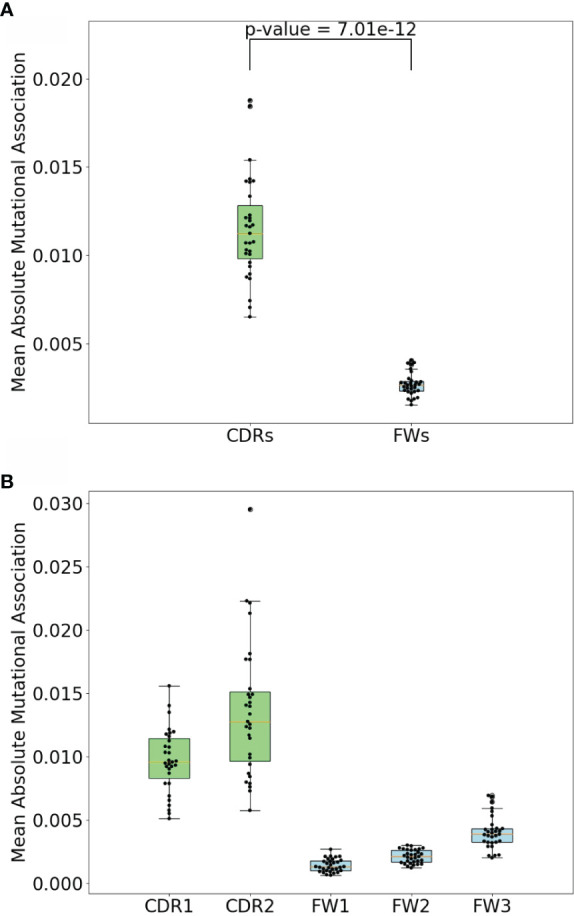
CDR vs FW MA comparisons. **(A)** Comparison of mean absolute MA for short-range dependencies between CDRs and FWs. P-value shown is a result. of Mann-Whitney (difference of means) test. **(B)** Comparison of mean absolute MAs within CDR1, CDR2, FW1, and FW2. Each dot represents a gene.

To compare the MA values in the different subregions, we calculated mean absolute MA values for distances 1 to 2 nt separately for the two CDRs and three FW subregions for all IGHV genes analyzed. As shown in **Figures 5A, B**, we indeed found that the CDRs had significantly higher absolute MA values. Because hotspot density is higher in the CDRs we surmised that short-range MAs, both positive and negative, might arise as a consequence of interactions between hotspots, i.e., mutations in one hotspot might affect the context and therefore the mutability of another hotspot. For example, consider the sequence AGCT, which has an overlapping AID hotspot (AGC) on both strands. If the bottom strand hotspot were to mutate, for example, from C to A (G to T on the top strand), this eliminates the top strand hotspot, since AGC has become ATC, which is a neutral site. Thus, the first mutation reduces the probability of the second site mutating (and vice versa if the mutations occur in the opposite order), which should lead to a negative correlation between the two sites. Such interactions between adjacent sites has long been recognized as a problem for producing genealogies because standard phylogenetic methods generally assume independence between sites (26). A recent study considered the mutations needed to produce three different broadly-neutralizing HIV antibodies (27). To quantify how likely it would be for these mutations to arise, the authors proposed a method (ARMADiLLO) that simulates mutations using a previously published set of substitution probabilities known as “S5F” (17). S5F substitution probabilities are defined for every nucleotide given its context of +/−2 nt, i.e., for every possible 5-mer of which there are 4^5^ = 1,024. Importantly, ARMADiLLO uses S5F to generate mutations one by one, changing the corresponding substitution probabilities to be used after each mutation.

We used the ARMADiLLO method to generate simulated datasets having the same distribution of mutations per sequence as the original data (mean mutation frequencies cannot be matched explicitly as these are defined internally by S5F). As shown in the orange curve of **Figure 3A**, ARMADiLLO largely recapitulates the initial peak corresponding to short-range MAs, underscoring the importance of sequential changes in substitution probabilities as each mutation occurs. ARMADiLLO performs less well on longer range MAs (> 2 nt distance), particularly when compared to the independent method. This suggests that longer-range features are primarily a statistical consequence of site-intrinsic high mutation frequencies, which are best replicated by the independent simulations. Naturally, this leads to the next question of what determines site-intrinsic mutability beyond local context (+/−2 nt) hotspots, which ARMADiLLO does consider. Although we will not address this question here, several potentially non-local (> 2 nt) mechanisms affecting SHM have been previously described, including DNA/RNA secondary structure (28), nucleosome phasing (29) and enhancer interactions (30), among others (31).

### A Generalized Overlapping Hotspot (OHS) Model

**Figure 6:**
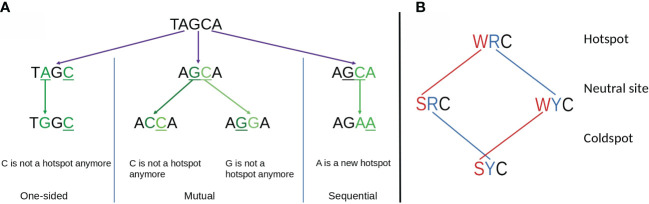
**(A)** Schematic showing three possible types of overlapping hotspots. Here using the example motif TAGCA: (left) One-sided overlap, (center) Mutual overlap, (right) Sequential overlap. **(B)** Effect of single mutations affecting hot, neutral, and cold AID mutation spots. At the first context nucleotide W to S mutations always reduce the hotness of the motif. Similarly, at the second position R to Y mutations always reduce the hotness of the motif. Both context nucleotides need to mutate to convert a hotspot into a coldspot.

To better explain the observed importance of short-range MAs and the relationship with hotspot interactions, we considered a generalized model of overlapping hotspots that took into account not only interactions between AID hotspots but also considered Polη hotspots. Each hotspot motif, for example AGC, contains a single nucleotide which we define as *mutating*, which in this example would be the underlined “C”, as well as one or more *context* nucleotides, which here would be “AG”. We can define an overlapping hotspot as containing two motifs such that the *mutating* nucleotide of one motif is, or becomes, a *context* nucleotide of the other. As illustrated with an example in **Figure 6A**, the overlap between the two hotspots could be either a: (a) one-sided overlap, as shown in **Figure 6A** (left), where the mutation of the Polη hotspot TA to TG affects the context nucleotide of the overlapping AGC hotspot, changing it to the non-hotspot motif GGC. The change is one-sided in that if the AGC hotspot were to instead mutate first, the TA hotspot would be unaffected. (b) mutual overlap, as shown in **Figure 6A** (middle). Here AGCA is an example of the WGCW overlapping AID hotspot motif described above, and where mutation of one hotspot can eliminate the hotspot on the opposite strand, although this need not necessarily occur—for example, a G>A mutation changes AGC to AAC which remains an AID hotspot. (c) sequential overlap, as shown in **Figure 6A** (right), where a C>A mutation in the context of AGCA creates a new AA that did not previously exist.

**Table 1** describes all possible overlaps of AID and Polη hotspots, allowing for both self- and cross-overlaps. As shown, there are only two possible mutual overlaps: WGCW (AID-AID) and TA (Polη-Polη). There are four reverse-complementary pairs of one-sided overlaps: WAA/TTW (Polη-Polη), WARC/GYTW, WAC/GTW, and TAC/GTA (Polη-AID). There are three reverse-complementary pairs of sequential overlaps: WACC/GGTW and WRCRC/GYGYW (AID-AID); WRCA/TGYW (AID-Polη). As an example, the colored dots in the bottom part of **Figures 4A, B** show the positioning of these overlapping hotspots in the human IGHV3-23 gene, here suggesting that most of the larger short-range MA values can be associated with an overlapping hotspot.

We reasoned that it should be possible to predict positive or negative MA based on the interactions between overlapping hotspots. The prediction is based on two observations:

1. Mutations of a *context* nucleotide that increase the “hotness” of a nearby *mutating* nucleotide should lead to a *positive* MA between these two substitutions. Conversely, mutations of a context nucleotide decreasing the “hotness” of a mutating nucleotide should lead to *negative* MA. This observation follows from the definition of MA. Consider two motifs, *x* and *y*. If a mutation at motif *x* increases mutability of motif *y* then *p(x,y)>p(x)p(y)*, leading to positive MA, whereas if a mutation at *x* decreases mutability of *y* then *p(x,y)<p(x)p(y)* and MA will be negative.
2. Out of the three possible mutations for a *context* nucleotide within a motif (hotspot, coldspot or, neutral site), one mutation does not alternate the “hotness” of the motif, while the other two change it in the same direction. Thus, for WA hotspots (where the context nucleotide is W = A/T) one mutation (A>T or T>A) preserves the hotspot, whereas the other two (W>C and W>G) convert it to a non-hotspot. For AID hotspots (WRC), as shown in **Figure 6B**, both context nucleotides must mutate so as to create a coldspot (SYC), since single mutations can only convert it to a neutral site. Thus, it is also impossible for any substitution (e.g., A > G) to change a neutral site to a hotspot and for a different substitution (e.g., A > T) in the same context nucleotide to change it to a coldspot.

### Predictions Based on the Generalized OHS Model Match Observed MA Values

Based on the two observations above, we can make predictions for the sign of the MA for all overlapping hotspots defined in **Table 2**. For one-sided overlaps, consider AAGC, an instance of a WARC motif, as an example where a Polη WA hotspot overlaps with an AID WRC hotspot. Here if the A site mutates, one of the possible mutations (A > T) will not eliminate the WRC hotspot (it will become TGC) whereas the other two mutations (A>G, A>C) will change it to a neutral site (observation 2). At the same time, if the initial mutation occurs in the AGC mutating site, this does not directly affect the mutability of the 5′ AA hotspot. In this example, because mutation of the AA hotspot can only reduce mutability of the AGC hotspot (and mutation of the AGC has no effect on the AA hotspot), we would expect a negative correlation, or MA, between the two mutating nucleotides (observation 1). Equivalent arguments can be made for any one-sided overlap and we therefore expect MA to be negative for all one-sided overlaps. For mutual overlaps, we also expect a (possibly stronger) negative MA, since the mutating nucleotides mutually reduce mutability. However, for sequential overlaps the situation is the opposite. Consider a sequential overlap such as TGCGC (an instance of WRCRC) where an AID TGC hotspot overlaps a potential hotspot (CGC). If the hotspot mutates, two mutations (C>A, C>T) will create the new hotspot (AGC or TGC) at the second C site, whereas with a C>G mutation it would remain a neutral site. By observation 2 above, if one substitution creates the second hotspot, another at the same position cannot create a coldspot. At the same time, mutation of the second C site (before or after it has become a hotspot), has no effect on the 5′ TGC hotspot. All sequential overlaps share these basic features and therefore we always expect a positive MA between the two relevant sites.

**Table 2:** Statistical analysis of MA for the pairs of nucleotides within an overlapping hotspot context and compared to non-context pairs.

	Motif	Prediction	OHS context			no OHS context	OHS context vs no OHS context		
			E (ohs)	P-value(Wilcoxon)	Adjusted p-value (BH)	E (no ohs)	E (ohs)-E (no ohs)	P-value (Mann-Whitney)	Adjusted p-value (BH)
Mutual	TA	negative	-0.02043	1.96E-32	3.14E-31	NA	NA	NA	NA
	WGCW	negative	-0.02734	1.21E-22	6.47E-22	-0.00333	-0.02400	8.87E-20	2.66E-19
One-sided	WAA	negative	-0.00352	1.31E-01	1.61E-01	-0.00242	-0.00110	1.53E-01	1.77E-01
	TTW	negative	-0.00124	3.16E-01	3.37E-01	0.00023	-0.00147	4.36E-03	5.46E-03
	WARC	negative	-0.03475	9.45E-11	1.89E-10	-0.00707	-0.02768	2.76E-10	5.18E-10
	GYTW	negative	-0.02131	5.54E-12	1.27E-11	-0.00214	-0.01917	3.13E-20	1.17E-19
	WAC	negative	-0.00095	2.96E-01	3.37E-01	0.00025	-0.00120	4.84E-01	4.84E-01
	GTW	negative	-0.00453	1.40E-03	1.86E-03	-0.00062	-0.00391	1.30E-03	1.77E-03
	TAC	negative	-0.00266	4.44E-01	4.44E-01	-0.00029	-0.00238	2.71E-01	2.90E-01
	GTA	negative	-0.02125	6.82E-15	2.18E-14	-0.00252	-0.01873	2.92E-26	4.38E-25
Sequential	WACC	positive	0.00483	3.28E-30	2.63E-29	0.00192	0.00291	9.74E-22	4.87E-21
	GGTW	positive	0.00182	1.80E-17	7.20E-17	0.00108	0.00074	6.16E-07	1.03E-06
	WRCRC	positive	0.01589	5.26E-08	8.41E-08	0.00302	0.01287	6.47E-18	1.62E-17
	GYGYW	positive	0.01531	2.61E-06	3.80E-06	0.00210	0.01321	3.96E-13	8.48E-13
	WRCA	positive	0.00690	5.08E-13	1.36E-12	0.00202	0.00488	9.32E-22	4.87E-21
	TGYW	positive	0.00310	1.63E-10	2.89E-10	0.00191	0.00119	8.59E-05	1.29E-04

The sign of the mean MA matches our prediction for all motifs [“E(ohs)” column] and the corresponding positive or negative shift is significant according to Wilcoxon signed rank test for all the motifs except for WAA, TTW, WAC, and TAC (shown in red). For all motifs, the shift also has the predicted direction [“E(ohs)-E(no ohs)” column] when compared to non-context pairs. WAC, TAC, and WAA are the only motifs for which this shift was not significant according to Mann-Whitney U test (highlighted in red). Motifs with non-significant comparisons (WAC, TAC, WAA, and TTW) are highlighted in yellow. Motifs with successful significant predictions are highlighted in blue.

We next sought to validate our prediction that mutual and one-sided overlaps lead to negative MA values, whereas sequential overlaps lead to positive MAs. As shown in **Table 2**, the observed MA values are all of the predicted sign and, for 12 of the 16 motifs, are statistically significant. To further confirm the importance of the context nucleotides in defining each motif, we compared the distribution of MA values for each motif (e.g., WGCW) to those motifs that have the same mutating nucleotides but different context nucleotides. For example, in the case of WGCW the three motifs that have the same mutating nucleotides (GC) but different context nucleotides are: SGCW, WGCS, and SGCS. We now considered the distribution of in-context MA values between the mutating nucleotides (G and C), specifically comparing the in-context MA distribution with the out-of-context MA distribution, with the expectation that the in-context MA values should be larger in the predicted direction. The comparative results (**Table 2**) show, that in-context motifs indeed have stronger MAs in the predicted direction than out-of-context MAs, and for all but three motifs, this difference is statistically significant.

### OHS Sites are Dominant for the Overall Pattern of MA Values

We recognize that in reality, correlations between mutation sites will be determined by a range of mechanisms and will be more complicated than this simple model of overlapping motifs suggests. Particular substitutions (e.g., transitions vs transversions) may have different probabilities, mutability for different motifs may be more heterogeneous than can be represented by neutral, hot- and cold-spots; also there is a dependency of Polη mutations on the original AID mutations, among other factors. Regardless, we sought to show how even such a simple model might be able to predict the majority of the strong MA signals. We therefore calculated MA values for all nucleotide pairs, of which there are 4^2^ = 16. This was done for adjacent (e.g., GC) and gapped (separated by one nucleotide which we will denote in the form “G_C”). Some nucleotide pairs may be able to have an overlapping motif (one-sided, mutual, or sequential), whereas others may not. Thus, for example, a subset of GC pairs will occur in the context of the mutual overlap motif WGCW; also, a subset of gapped A_C pairs will be one-sided overlaps with the motif WARC (**Table 1**). Note that each nucleotide pair, adjacent or gapped, can only occur in the context of at most one overlap type. We reasoned that if the overlap motif (e.g., WGCW) is the major determinant of MA for the nucleotide pair within (e.g., GC), then pairs that can contain negative MA motifs (one-sided and mutual) should have negative MA on average. Similarly, nucleotide pairs compatible with sequential motifs should have positive MA on average. **Table 3** shows the results for all nucleotide pairs, both adjacent and gapped, ordered by mean strength, defined here as the deviation from mean overall MA. As shown in **Table 3**, the predicted sign of the MA values match those observed for all overlap motifs. Furthermore, almost all overlap motifs (12 of 16) are in the top half of the table in terms of strength. The results of **Tables 2** and **3** suggest that overlap motifs are the dominant effect for most nucleotide pairs that allow an overlap motif. Overlap motifs are also dominant overall (**Table 3**) since most nucleotide pairs that cannot contain overlap motifs, also do not have strong MA values.

**Table 3:** Statistical analysis of the local MA for all possible pairs of nucleotides.

Pair of nucleotides	Deviation from mean MA	Overlapping HS			Predicted MA (sign)	Experimental MA	P-value(Wilcoxon)	Adjusted p-value
		One-sided	Mutual	Sequential				
TA	0.02037		TA		negative	-0.02097	8.55E-34	2.28E-33
GC	0.01366		WGCW		negative	-0.01426	1.23E-22	2.47E-22
A_C	0.01051	WARC			negative	-0.01111	1.64E-52	7.48E-52
G_A	0.00504	GTA			negative	-0.00565	3.83E-38	1.36E-37
G_T	0.00470	GYTW			negative	-0.00531	7.63E-34	2.22E-33
A_A	0.00408					0.00348	6.73E-35	2.15E-34
AG	0.00397					0.00337	2.73E-24	5.83E-24
CC	0.00386			WRCC	positive	0.00325	1.47E-79	1.56E-78
CA	0.00291			WRCA	positive	0.00231	1.00E-56	8.02E-56
C_C	0.00280			WRCRC	positive	0.00219	9.98E-55	6.38E-54
GG	0.00273			GGYW	positive	0.00213	9.02E-104	2.89E-102
AA	0.00258	WAA			negative	-0.00318	1.29E-01	1.48E-01
G_G	0.00206			GYGYW	positive	0.00145	3.40E-53	1.81E-52
T_T	0.00179					0.00119	8.70E-12	1.64E-11
GA	0.00179					0.00118	1.28E-30	3.16E-30
GT	0.00170	GTW			negative	-0.00230	7.53E-08	1.34E-07
G_C	0.00160					0.00100	2.04E-84	3.27E-83
A_T	0.00146					0.00085	1.82E-02	2.42E-02
TC	0.00144					0.00084	3.27E-39	1.31E-38
T_G	0.00132					-0.00192	1.30E-04	2.08E-04
TG	0.00127			TGYW	positive	0.00066	4.45E-30	1.02E-29
T_A	0.00126					0.00066	3.97E-02	4.89E-02
AT	0.00105					-0.00165	7.22E-02	8.56E-02
C_T	0.00077					0.00016	2.45E-02	3.13E-02
T_C	0.00076	TAC			negative	-0.00136	7.07E-05	1.19E-04
C_A	0.00072					-0.00132	7.40E-03	1.13E-02
A_G	0.00050					-0.00011	8.99E-03	1.25E-02
CT	0.00044					-0.00016	9.16E-01	9.16E-01
C_G	0.00030					-0.00031	4.04E-01	4.31E-01
CG	0.00028					-0.00032	7.86E-03	1.14E-02
TT	0.00021	TTW			negative	-0.00081	5.01E-01	5.17E-01
AC	0.00019	WAC			negative	-0.00079	2.44E-01	2.70E-01

Data is sorted based on the deviation from the overall mean MA. Pairs with predicted negative MA are highlighted in blue, positive predictions are highlighted in red. P-values represent significance of the shift between original data MAs and those from the Independent simulations. Non-significant p-values are highlighted in purple, less significant p-values are highlighted in light purple.

As a further test of the importance of generalized overlapping hotspots, we evaluated a simple overlapping hotspot model, as follows. Using the germline IGHV alleles in our original dataset, for each pair of nucleotides that are either adjacent or one nucleotide apart, we assigned a +1 to pairs with positive predicted MA, −1 to those with predicted negative MA (matching the column “predicted MA” of **Table 3**) and 0 to all other pairs. We then compared these sign values to the corresponding MA values in the original dataset and found a modest, though highly significant, correlation (Pearson r = 0.25, P < 10^−50^). When we repeated the comparison using the corrected dataset, the correlation went up to r = 0.37 (P < 10^−50^). When we used the short-range MA values derived from the ARMADiLLO simulations used for **Figure 3**, again comparing to the original dataset MAs, we found a very similar correlation (Pearson r = 0.24, P < 10^−50^; r = 0.25, P < 10^−50^, for corrected versions of ARMADiLLO and original data). At the same time, the independent simulations gave a small negative correlation (r = −0.05) with the original dataset MAs. In summary, an extremely simple model, based only on generalized overlapping hotspots and the predicted sign of their MA value, performs at least as well on this task, compared to a far more complicated simulation model (ARMADiLLO) that uses S5F mutabilities.

**Figure 7:**
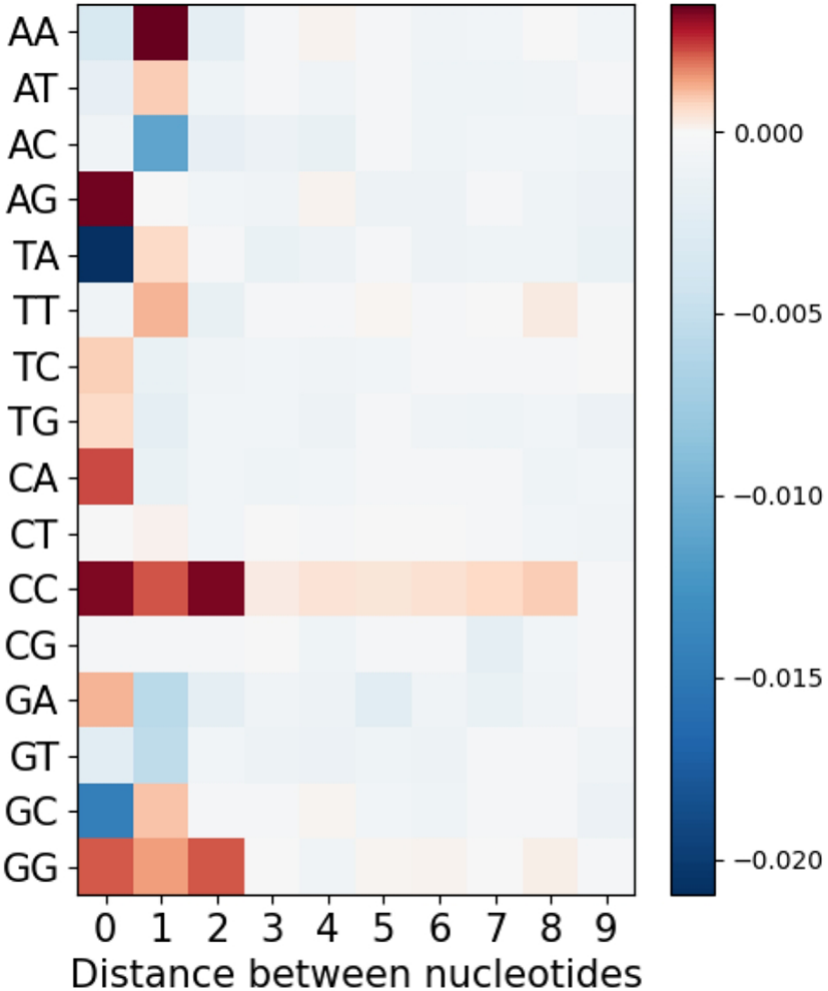
Mean MA for all possible pairs of nucleotides separated by different distances from each other.

Lastly, we considered longer-range MA values by extending the analysis of **Figure 3**, only now separating the different nucleotide pairs, to evaluate whether there might be longer-range effects specific to some nucleotide pair, but that are weakened by averaging in **Figure 3**. We therefore considered not only adjacent and single-gap nucleotide pairs, but also bigger gaps from 2 to 10 nt. **Figure 7** shows mean MA values as a heatmap for all nucleotide pairs (rows) and with different gap sizes (columns) between them. As we might expect, the results are largely consistent with **Figure 3**. However, we did notice some longer-range effects for pairs of C or G nucleotides. The simplest explanation for these would be AID processivity (see Discussion).

## Discussion

Somatic hypermutation is driven by a biased mutation process that strongly favors particular patterns of mutations in the IgV genes, particularly in certain subregions such as the CDRs (10). Following the discovery of AID and the associated mechanisms of ncBER and ncMMR, the hotspots for AID (motif WRC) and Polη (motif WA) were characterized (16, 32) and also found to be more common in highly mutated subregions such as the CDRs. Previous work that compared nonproductive IGHV sequences (which never underwent selection) with productives (that presumably did) found the mutational spectra to be almost identical (12), suggesting that the underlying mutational bias is strong enough to dominate over selection effects. Several previous studies have addressed the intrinsic mutability of a site as a function of the immediate neighboring sites, for example, the S5F model (17) that describes mutability scores based on the surrounding +/−2 nt. Here we focused on a different but related issue: how mutations at one site might influence mutations at another site. We defined mutational association (MA) as a measure of correlation between sites and used it to analyze pairwise site correlations within a large database of high quality UMI-barcoded human IGHV sequences. Two distinct effects were clearly observable: a strong short-range (1–2 nt) correlation effect, and a weaker longer-range effect at inter-site distances greater than 2 nt.

High intrinsic mutation frequencies at pairs of sites are expected to lead to some degree of correlation. The observed short-range correlations were far higher than expected when compared to simulations using only site-intrinsic mutation frequencies, although these “Independent” simulations (**Figure S2A**) did largely explain the longer-range correlations (**Figure 3A**). Our results suggested that short-range correlations were caused by mutations that alter the mutability of adjacent sites during the course of SHM. Simulations using the ARMADiLLO method that allow mutations to change the context (and therefore the mutability) of nearby sites as SHM proceeds, were indeed able to largely recreate the short-range correlations that were missing from the “Independent” simulations (though they showed worse performance describing the overall MA landscape, see **Figure 3A**). Our results suggested that interactions between AID and/or Polη hotspots might largely explain the short-range correlations.

Previous work by ourselves and others had studied the WGCW motif, which contains two overlapping AID hotspots on opposite strands, and which tends to mutate particularly highly (6, 7). In order to better explain the short-range correlations, we sought to generalize the concept of overlapping hotspots to include Polη (WA) hotspots, also accounting for sequential overlaps, where a mutation in one hotspot may create another hotspot that did not exist previously. The WGCW motif is an instance of a mutual overlapping hotspot, as is TA, which contains a WA hotspot on both strands. However, in several cases Polη and AID hotspots form one-sided overlaps such that mutation of one hotspot can eliminate the other, but not the other way around. Surprisingly, simply using the predicted sign for the overlapping hotspot was as predictive of MA as using the far more complicated ARMADiLLO method.

The sequences used in our analysis were purposefully chosen from different clonal groups to ensure the independence of the mutations therein. To extend our results a little further we sought some direct evidence for our MA predictions within, rather than between, clones. If, for example, we consider a pair of sites, such as AGCT (an instance of the WGCW motif), and then compare sequences derived from an ancestor with a mutation at one site (which we will refer to as “Site 1”, e.g., the G in AGCT), to sequences without that mutation, then we would expect the C in the AGCT (“Site 2”) to mutate more often in the latter case (unmutated G), consistent with the predicted negative MA. However, it is also necessary to control for the number of mutations between ancestral and derived before making such a comparison. To test this approach, we analyzed two AGCT sites (at positions 106 and 162) within IGHV3-23*01 that are highly mutated and that were the focus of a previous study (7), where the sites were labeled as “OHS1” and “OHS2”, respectively (**Table S1**). Considering the G as “Site 1” first (and comparing the mutation frequency of the C as “Site 2” across different clones), then the opposite condition (C as “Site 1”, G as “Site 2”), leads to two conditions for each of the two sites. Because of the negative predicted MA, we expect the “Site 2” mutation frequency to be lower when “Site 1” is mutated. A total of 41 comparisons were made at distances up to 14 mutations (**Table S1**), of which 38/41 = 93% were in the predicted direction and, of those, 33/38 = 87% were statistically significant (FDR corrected P <0.05). We further confirmed that the mean direction was significant for all conditions except OHS1/C-G, which only had four data points (**Table S1**).

**Figure 8:**
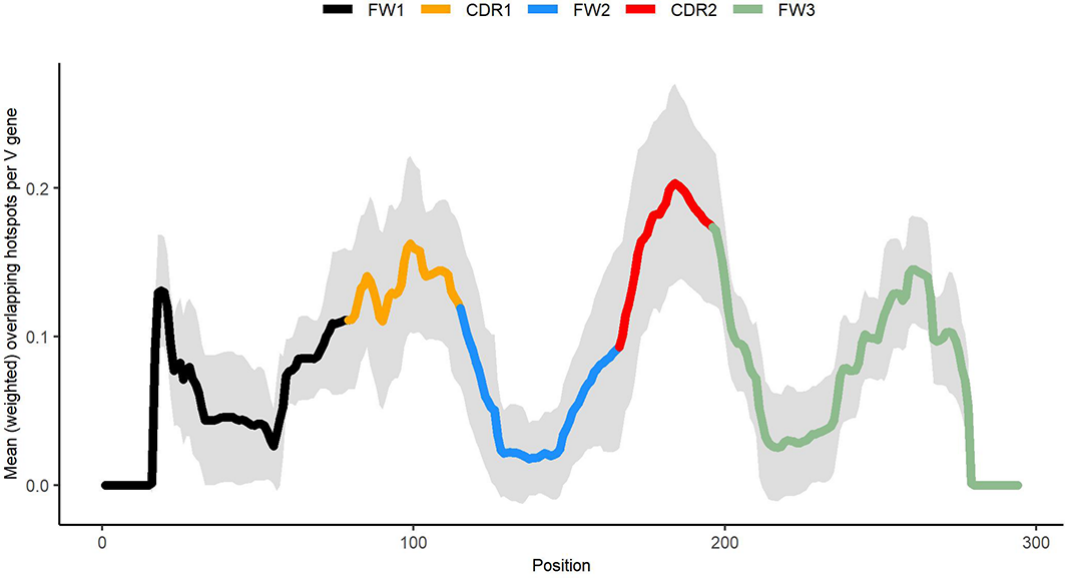
Profile describing abundance of generalized overlapping hotspots. Densities for the hotspots of **Table 1** added together and weighted according to expected abundance (see *Materials and Methods*).

As shown in **Figure 8**, generalized overlapping hotspots are more abundant in highly mutating subregions such as the CDRs, although there are substantial densities also at the 5′ end of FW1 and within a subregion of FW3 often referred to as “CDR4” (25), which is a pattern similar to that found in our previous analysis that considered only WGCW and WA hotspots (9). Although to an extent this is expected, given the higher density of hotspots in CDRs, the fact that CDR mutations are subject to strong local correlations shows that these key mutations are far less random than previously appreciated. We found that overlapping hotspots explain almost all significant correlations between nucleotide pairs, whether adjacent or with gaps. The only exception was the positive correlations between C nucleotides (and to a lesser extent, Gs) mutations on the same strand, which may be explained by AID processivity. Previous biochemical studies have described AID processivity as acting by a jump and slide mechanism both on naked ssDNA and on transient ssDNA during transcription (33, 34). Although one previous analysis did suggest there was processivity *in vivo* (22), the analysis we present here is the first, to our knowledge, that shows a mutation pattern consistent with processivity *in vivo* arising from analysis of high throughput human IGHV data. At the same time, very few nucleotide pairs had highly significant MA values but did not fit into the generalized overlapping hotspot scheme. For example, A adjacent to G (“AG”, see **Table 3**) or “A_A” were both significantly positive. In both cases, MA appeared to be stronger if one or both sites are hotspots, even if here they are adjacent (non-overlapping) hotspots. For example, if we considered “AG” in the context of the motif TAGYW and compare it to non-context AG pairs (as was done for **Table 2**), we find the in-context AG pairs to be significantly higher (in-context MA: 0.047, non-context MA: 0.008, Mann-Whitney P = 3.05e-08). Similarly, for “A_A”, the difference is higher if the second site is a hotspot, i.e., ATA (in-context MA: 0.032, non-context MA: 0.013, Mann-Whitney P = 4.78e-10).

Several mechanisms have previously been proposed that potentially lead to multiple simultaneous mutations and, therefore, to positive MA and possibly between relatively distant mutation sites (35–37). In our analysis of productive sequences, we do not observe any systematic evidence of such mechanisms—**Figure 7** shows no evidence for such associations besides local ones, with the possible exception of AID processivity discussed above. However, multiple simultaneous mutations are likely to be filtered out by selection, since if more mutations occur at once, it is more likely that at least one of them will be disadvantageous. In the analysis of nonproductive sequences we observe a slight increase in non-local signals (see **Figure S6A**), which would be consistent with simultaneous mutations that are not being selected against, although more noise is also expected due to having less non-productive data.

Although most short-range MAs could be explained in terms of the generalized OHS model, one of the strong local signals which could not was the strong positive association between A and G, when A is immediately followed by G. This particular case may however be explained in terms of the model proposed by Thientasapol et al. (37). In this study, the authors propose a mechanism following an original C mutation in the context of an AGCT, where one of the possible outcomes involves Polh recruited to the strand opposite from the original mutation. Here, the original mutation is not repaired by Polh while at the same time new Polh mutation(s) may be introduced to the opposite strand, leading to simultaneous AID and Polh mutations and thus, to positive MA. Consistent with this mechanism, we observe a particularly strong positive association between A and G in a TAGYW context (adjacent Polh and AID hotspots on opposite strands). Since we observe this effect in a more general context than AGCT, it suggests that this mechanism may be relevant to other WGCW motifs and possibly even to regular AID hotspots. At the same time, because the effect is only clear for a Polh hotspot immediately adjacent to an AID hotspot, the effect may decrease rapidly with the distance from original mutation with more distant occurrences being too infrequent to overcome selection. Furthermore, since this effect is observed for A and G, but not for C and T, this suggests a potential strand bias.

Our results concerning overlaps between AID and Polη hotspots also give us some clues as to why these particular hotspot motifs (WRC, WA) have co-evolved and, as far as we know, have remained evolutionarily stable throughout the jawed vertebrates that use AID for somatic hypermutation (38). Polη is a highly conserved enzyme in eukaryotes which also functions during canonical DNA damage repair, for example in response to sunlight UV-B radiation DNA damage (39). In contrast, AID functions almost exclusively during SHM and we know that, at least in principle, its hotspot preference did not have to remain as conserved because other members of the closely-related AID/APOBEC cytidine deaminase family have evolved distinct hotspots (40). Thus, it appears that AID and Polη hotspots may be optimally suited to one another. For example, as shown in **Table 1**, interactions between WA/TW and WRC motifs lead to three possible one-sided overlaps and one sequential overlap. In particular, WA can overlap a WRC motif both at the first context nucleotide (W**A**RC), at the second (A**A**C) or both (**TA**C). Furthermore, as noted in the Results, any individual mutation in a context nucleotide of WRC cannot lead directly from a hotspot to a coldspot, suggesting the WA and WRC hotspots may have co-evolved to generate gradual changes in mutability. Another key function for AID is in class switch recombination (15). Several previous studies have found that WGCW motifs such as AGCT are particularly highly mutated in both V and switch regions, leading to DSBs in switch regions and quite possibly in V regions also (41). The need for double-stranded breaks (DSBs) may constrain the second context nucleotide (W**R**C) to allow a G for this purpose, as it does (R = A/G). Indeed, the WRC motif is optimal among all the possible αβC trimers (where α and β allow for two nucleotides each), in terms of allowing overlaps with Polη hotspots while also allowing potential DSBs (**Table S2**). From the perspective of Polη overlaps, the motifs WMC and WWC are also good, but neither of them contains a GC motif to facilitate DSBs. Recent work by ourselves showed that co-localization of WGCW and WA hotspots further appears to be a defining feature of the CDRs across all human IGHV genes (9), which may also be explained by their natural compatibility.

The generalized overlapping hotspots we have introduced so far (**Table 2**) have a further potential to overlap with each other, leading to a large number of composite motifs. For example, consider the sequence GGTAGCAC situated at the 3′ end of CDR2 within the IGHV3-23*01 allele (see **Figure 4**). Here, eight different overlapping hotspot motifs (GGTA, GTA, GTA, TA, TAGC, AGCA, AGCA, AGCAC) are packed within just eight nucleotides (see **Table S3**). Furthermore, all of the predictions made by our overlapping hotspot model for these motifs fit the observed MA values (**Figure S4**).

## Data Availability Statement

Publicly available datasets were analyzed in this study. This data can be found here: https://www.ncbi.nlm.nih.gov/bioproject/?term=381394 https://www.ncbi.nlm.nih.gov/bioproject/?term=591804.

## Author Contributions

AK and TM conceived the idea, designed models and methods, analyzed results, and wrote the manuscript. AK and CT performed computational analysis. All authors contributed to the article and approved the submitted version.

## Funding

This work was supported by grant NIH R01AI132507 to TM. The funders had no role in study design, data collection, and interpretation or the decision to submit the work for publication.

## Conflict of Interest

The authors declare that the research was conducted in the absence of any commercial or financial relationships that could be construed as a potential conflict of interest.
