# Supplementary material for: Correlations in Somatic Hypermutation Between Sites in IGHV Genes Can Be Explained by Interactions Between AID and/or Polη Hotspots
Source: Front Immunol. 2021 Feb 2;11:618409. doi: 10.3389/fimmu.2020.618409 (PMC7884765; doi:10.3389/fimmu.2020.618409)
Supplement: Supplementary file 1 [file DataSheet_1.docx]

**Supplementary materials**


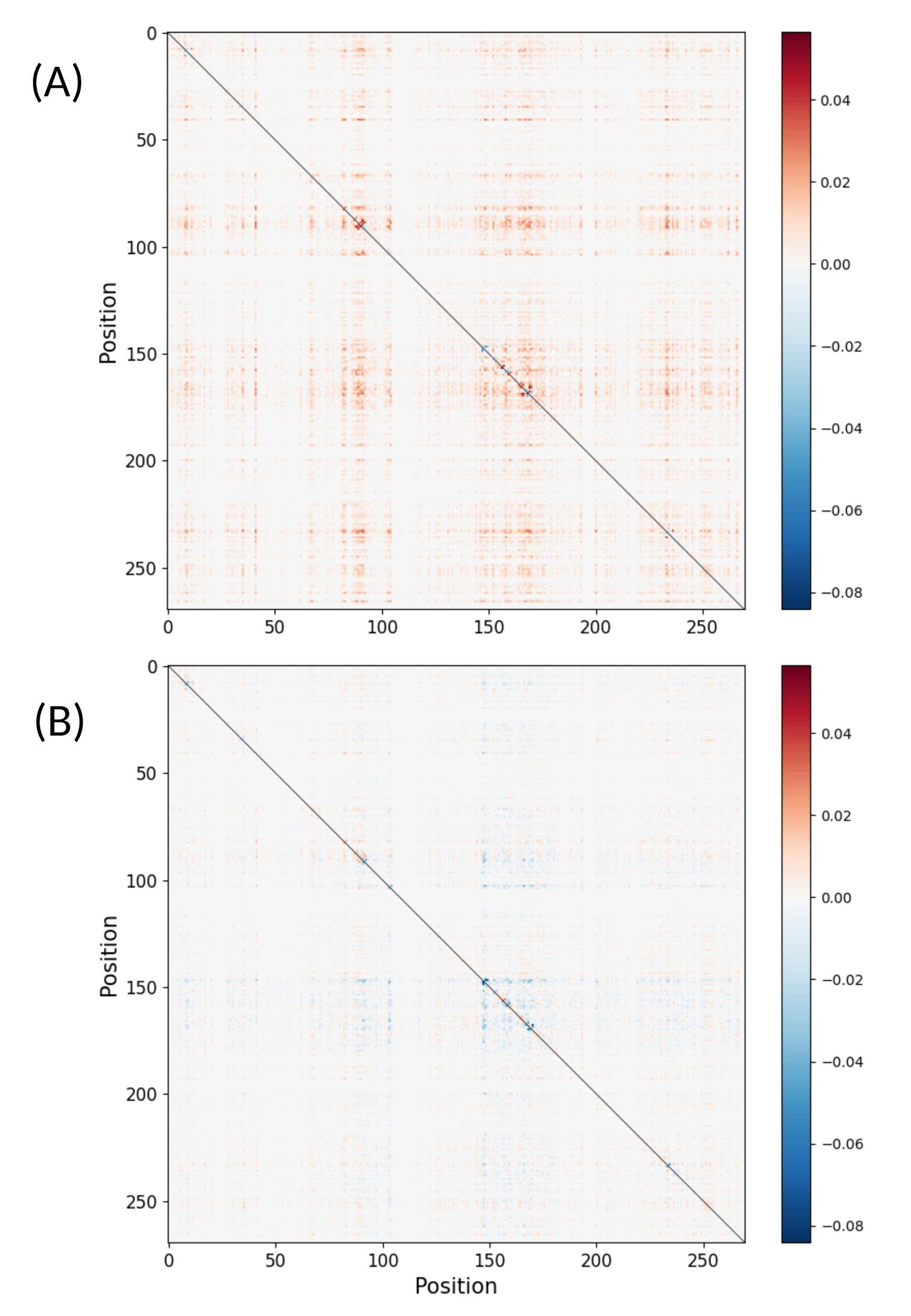


**Supplementary Figure 1.** MA heatmaps for human IGHV3-23*01. **(A)** MAs for the original data without correction, **(B)** corrected MAs, result of subtraction of MAs for Independent dataset (See Fig S2A) from original data MAs.

**
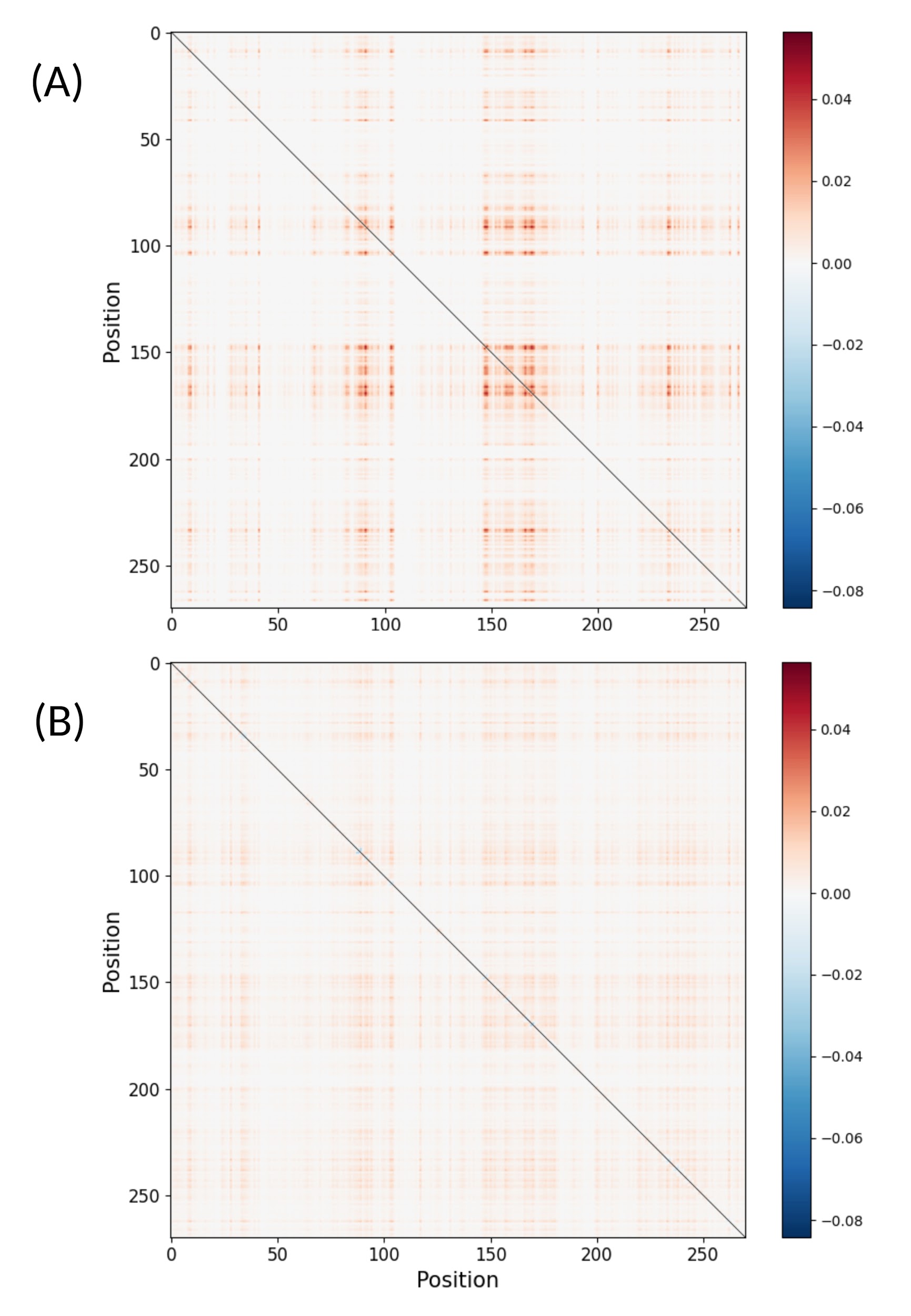
**

**Supplementary Figure 2.** MA heatmaps for human IGHV3-23*01. **(A)** MAs for Independent simulated dataset, **(B)** MAs for ARMADiLLO dataset.

**
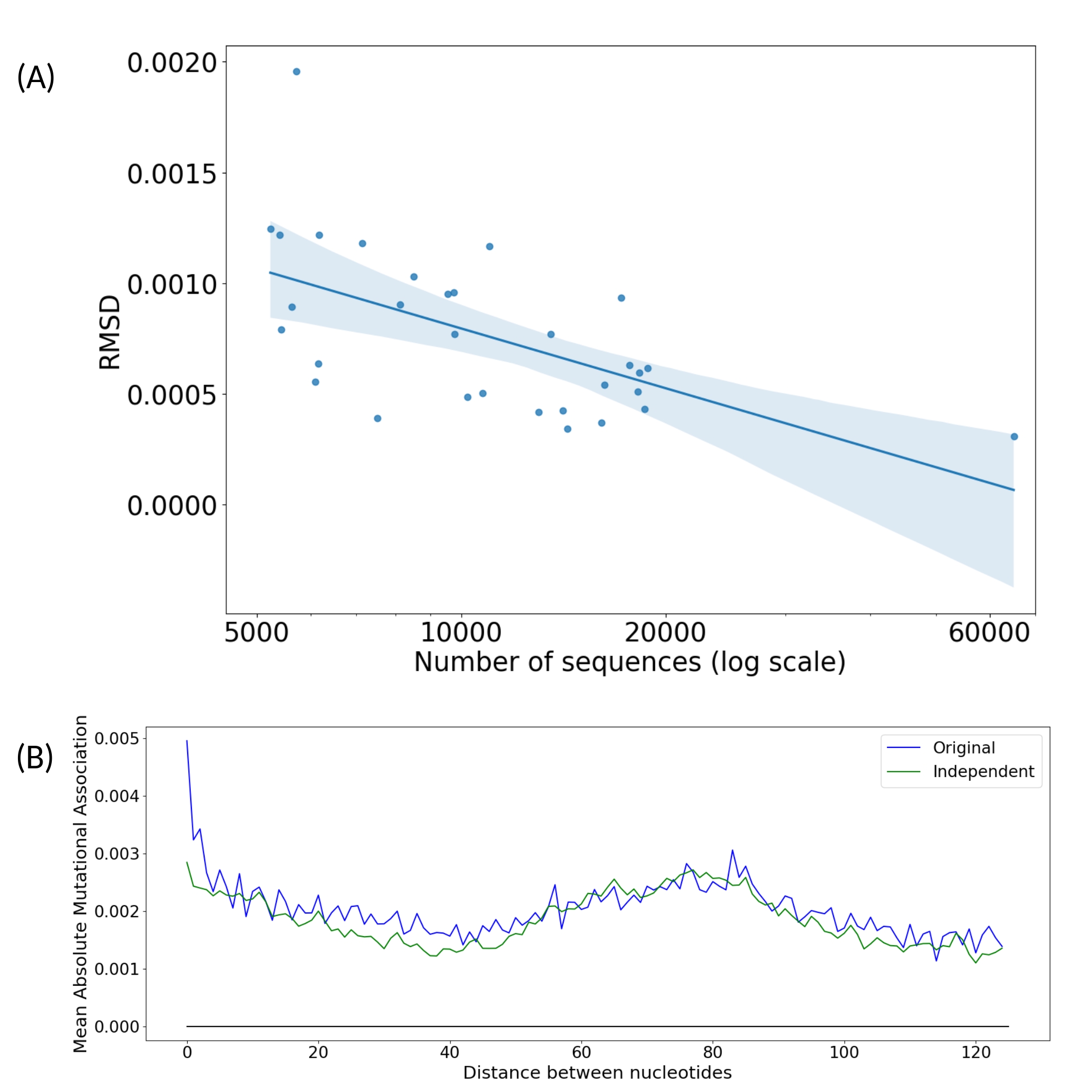
**

**Supplementary Figure 3.** Sampling bias in independent simulations. The gap between Original and Independent lines (in plots equivalent to Figure 3A but for separate alleles), as measured by root mean squared distance (RMSD), decreases when the amount of data for a given allele increases. **(A)** Scatter plot showing dependence of the RMSD between the original and independent lines, and number of sequences (log base 10), for the 31 alleles analyzed. **(B)** Plot equivalent to Figure 3A for the allele we have the most data for (IGHV3-23*01, 65071 sequences). Here, there is essentially no gap between the original and independent lines, except for the shortest distances, as expected.

**
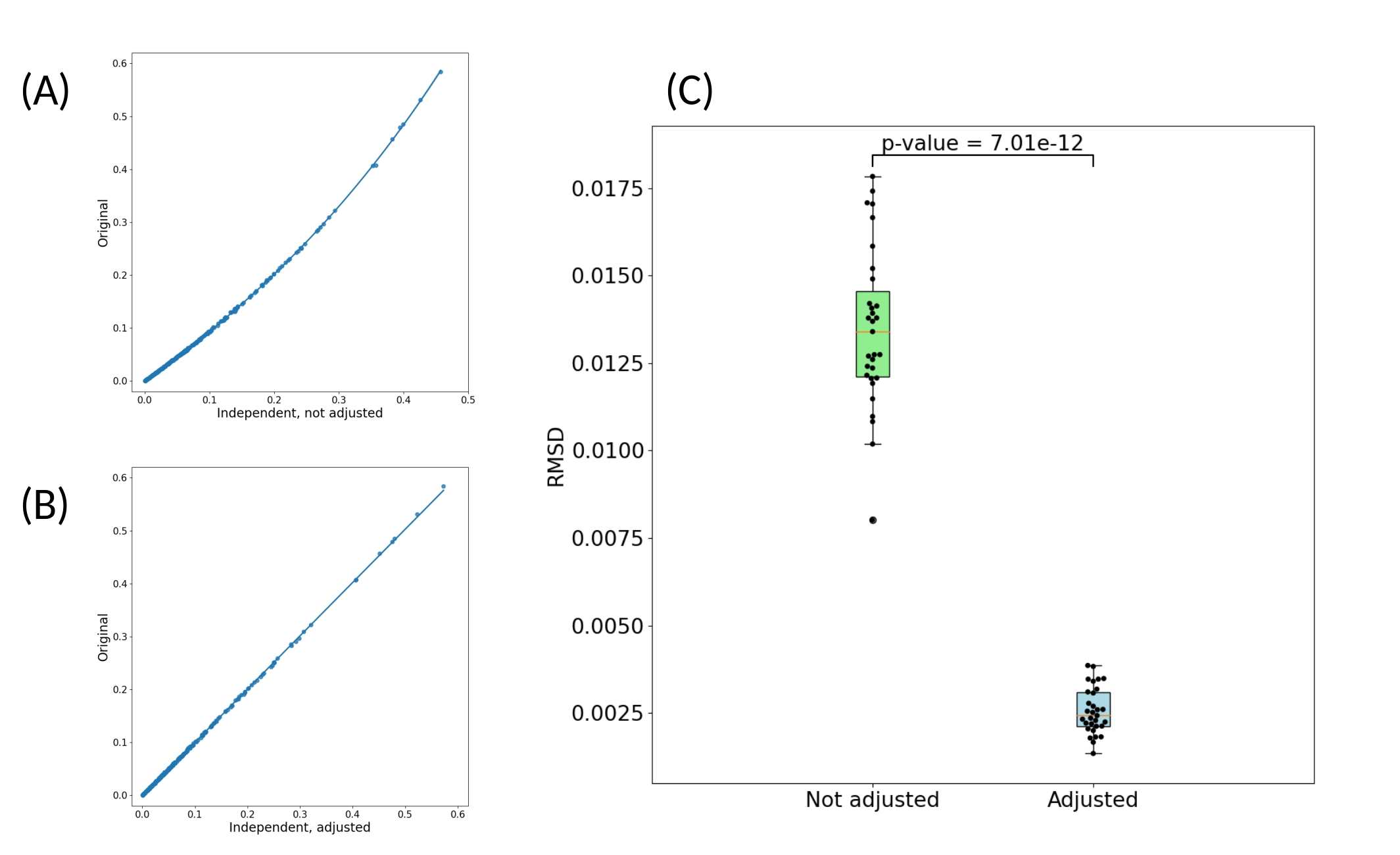
**

**Supplementary Figure 4.** Comparison of the accuracy of the Independent simulation mutation frequencies with and without cubic adjustment. **(A)** Independent mutation frequencies vs Original mutational frequencies without cubic adjustment, together with cubic regression curve, each dot represents a mutation site, **(B)** Independent mutation frequencies vs Original mutational frequencies after cubic adjustment for IGHV3-23*01, each dot represents a mutation site, **(C)** RMSD of Independent mutation frequencies from the Original mutational frequencies with and without cubic adjustment, showing one dot per allele.

**
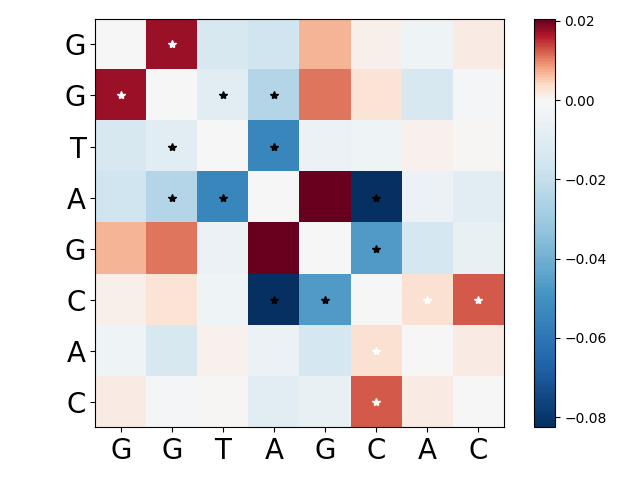
**

**Supplementary Figure 5.** Observed MA values for the GGTAGCAC motif at the 3’ end of CDR2 in the IGHV3-23*01 allele. Asterisks indicate positions of overlapping hotspots as detailed in Table S1. White asterisks correspond to sequential overlaps (positive prediction), black asterisks correspond to one-sided and mutual overlaps (negative prediction).

**
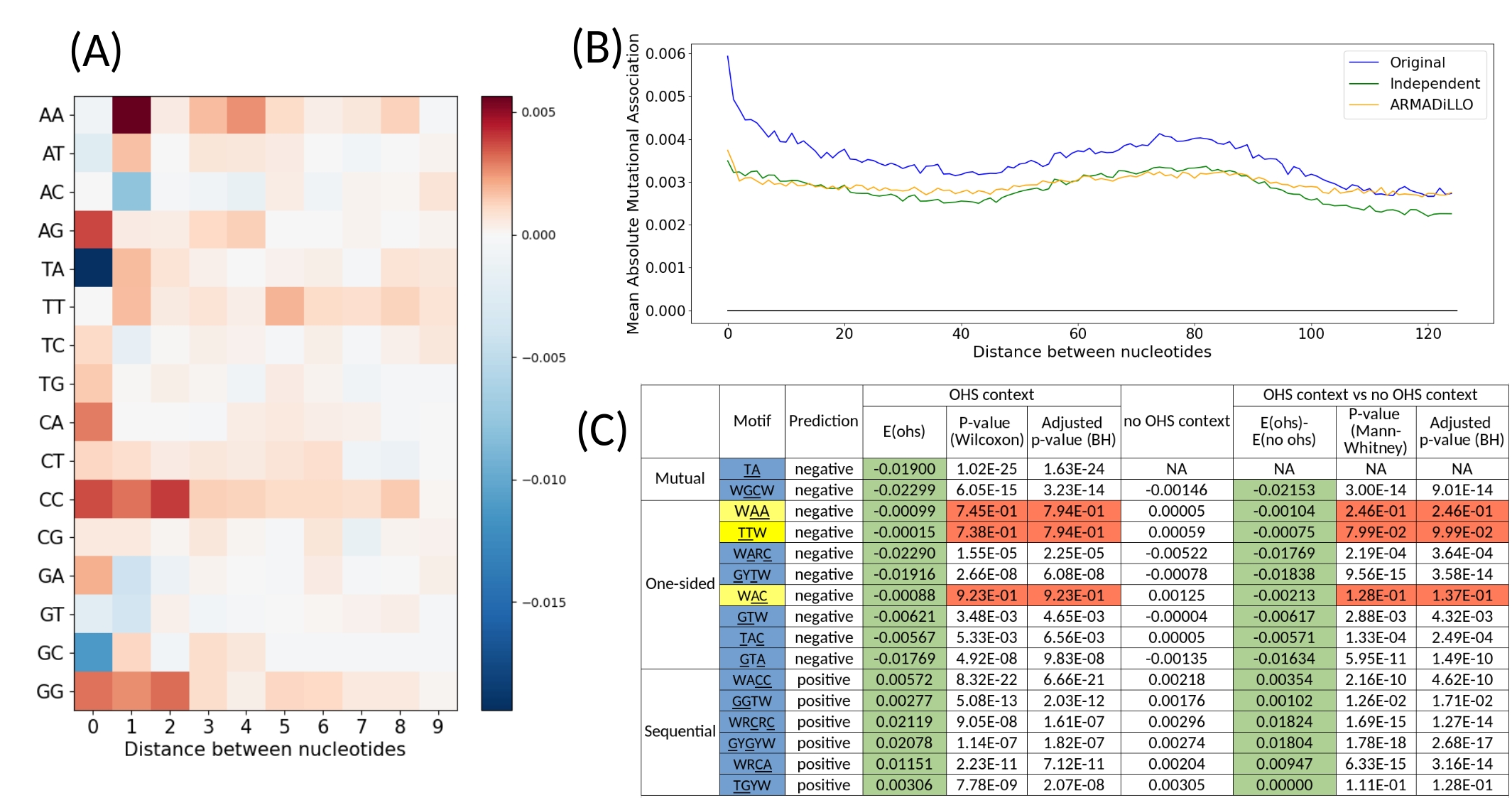
**

**Supplementary Figure 6.** Main results recreated using nonproductive data. **(A)** Equivalent to Figure 7, **(B)** Equivalent to Figure 3a, **(C)** Equivalent to Table 2.

**
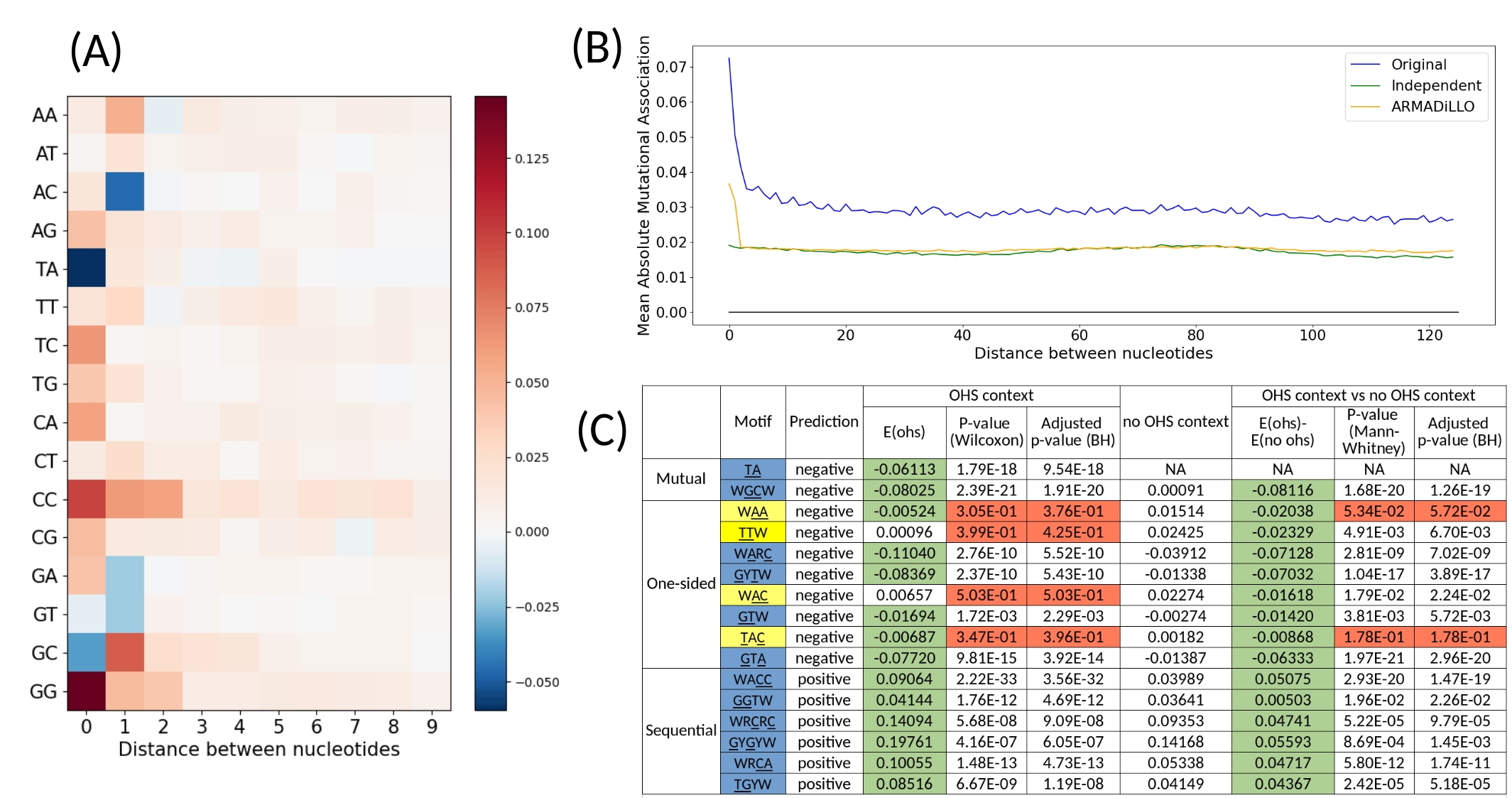
**

**Supplementary Figure 7.** Main results recreated using Pearson correlation measure instead of MA. **(A)** Equivalent to Figure 7, **(B)** Equivalent to Figure 3a, **(C)** Equivalent to Table 2.

**
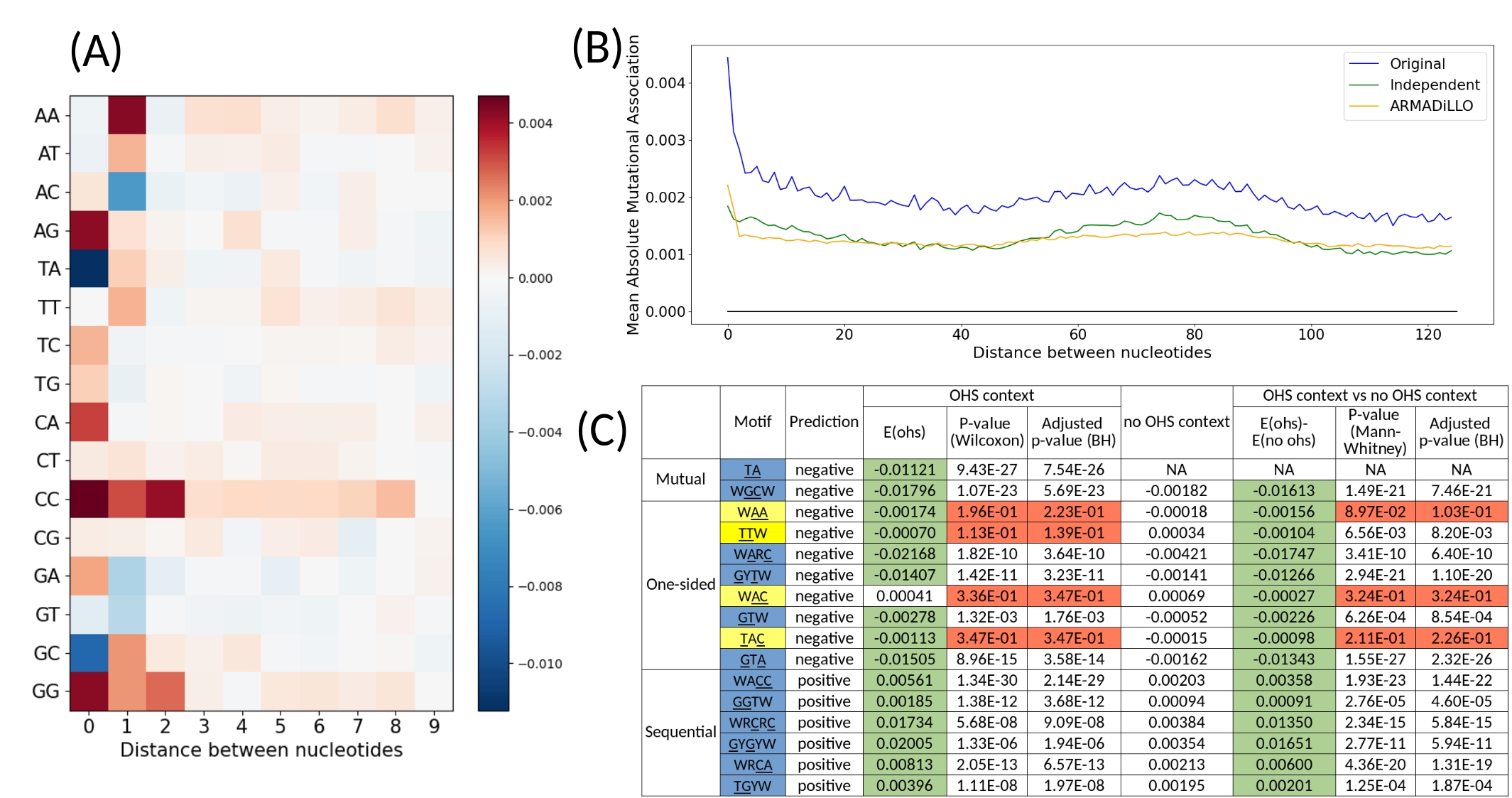
**

**Supplementary Figure 8.** Main results recreated using LD metric instead of MA. **(A)** Equivalent to Figure 7, **(B)** Equivalent to Figure 3a, **(C)** Equivalent to Table 2.

| distance  from the ancestral sequence | OHS1 | | | | | | | | | |
| --- | --- | --- | --- | --- | --- | --- | --- | --- | --- | --- |
|  | OHS1/G-C | | | | | OHS1/C-G | | | | |
|  | shift | # of clones in control group | # of clones in case group | p-value | adjusted  p-value | shift | # of clones in control group | # of clones in case group | p-value | adjusted  p-value |
| 1 | -0.00005 | 870 | 1221 | 4.01E-03 | 1.21E-02 | -0.00891 | 908 | 304 | 7.79E-02 | 8.40E-02 |
| 2 | -0.02191 | 253 | 357 | 3.65E-04 | 2.99E-03 | -0.03983 | 276 | 75 | 3.99E-02 | 4.54E-02 |
| 3 | -0.01965 | 152 | 190 | 7.04E-04 | 4.12E-03 | -0.10641 | 167 | 35 | 9.46E-02 | 9.69E-02 |
| 4 | -0.04697 | 98 | 144 | 5.89E-04 | 4.02E-03 | -0.18837 | 113 | 17 | 1.49E-01 | 1.49E-01 |
| 5 | -0.06013 | 79 | 107 | 8.86E-04 | 4.39E-03 | NA | NA | NA | NA | NA |
| 6 | -0.00226 | 66 | 63 | 4.13E-03 | 1.21E-02 | NA | NA | NA | NA | NA |
| 7 | 0.01997 | 79 | 53 | 8.79E-03 | 1.70E-02 | NA | NA | NA | NA | NA |
| 8 | -0.05150 | 49 | 29 | 1.16E-02 | 1.70E-02 | NA | NA | NA | NA | NA |
| 9 | -0.12438 | 45 | 29 | 5.56E-03 | 1.34E-02 | NA | NA | NA | NA | NA |
| 10 | -0.05714 | 44 | 22 | 8.40E-03 | 1.70E-02 | NA | NA | NA | NA | NA |
| 11 | -0.14286 | 28 | 14 | 9.80E-03 | 1.70E-02 | NA | NA | NA | NA | NA |
| 12 | -0.43623 | 23 | 15 | 9.63E-04 | 4.39E-03 | NA | NA | NA | NA | NA |
| 13 | -0.23942 | 18 | 23 | 3.91E-02 | 4.54E-02 | NA | NA | NA | NA | NA |
| distance  from the ancestral sequence | OHS2 | | | | | | | | | |
|  | OHS2/G-C | | | | | OHS2/C-G | | | | |
|  | shift | # of clones in control group | # of clones in case group | p-value | adjusted  p-value | shift | # of clones in control group | # of clones in case group | p-value | adjusted  p-value |
| 1 | -0.00229 | 734 | 713 | 1.30E-05 | 5.33E-04 | -0.00696 | 732 | 848 | 1.42E-04 | 1.46E-03 |
| 2 | -0.00111 | 222 | 203 | 2.95E-05 | 6.04E-04 | -0.03894 | 217 | 215 | 1.37E-04 | 1.46E-03 |
| 3 | 0.01365 | 111 | 121 | 4.91E-03 | 1.34E-02 | -0.09942 | 114 | 101 | 1.70E-02 | 2.25E-02 |
| 4 | -0.03485 | 97 | 74 | 5.90E-03 | 1.34E-02 | -0.11723 | 101 | 74 | 1.07E-02 | 1.70E-02 |
| 5 | 0.01914 | 67 | 67 | 3.45E-03 | 1.21E-02 | -0.21417 | 70 | 52 | 9.86E-03 | 1.70E-02 |
| 6 | -0.04077 | 57 | 47 | 1.15E-02 | 1.70E-02 | -0.14017 | 59 | 41 | 7.13E-02 | 7.90E-02 |
| 7 | -0.04068 | 63 | 37 | 1.26E-02 | 1.78E-02 | -0.23420 | 62 | 33 | 3.39E-02 | 4.21E-02 |
| 8 | -0.15857 | 36 | 21 | 7.04E-03 | 1.52E-02 | -0.38003 | 41 | 23 | 3.62E-03 | 1.21E-02 |
| 9 | -0.16864 | 33 | 23 | 1.00E-02 | 1.70E-02 | -0.36679 | 33 | 16 | 2.55E-02 | 3.26E-02 |
| 10 | -0.32258 | 31 | 18 | 3.93E-03 | 1.21E-02 | -0.24167 | 32 | 15 | 5.48E-03 | 1.34E-02 |
| 11 | -0.16071 | 21 | 16 | 1.13E-02 | 1.70E-02 | NA | NA | NA | NA | NA |
| 12 | -0.22308 | 20 | 13 | 1.69E-02 | 2.25E-02 | NA | NA | NA | NA | NA |
| 13 | -0.16188 | 12 | 11 | 9.29E-02 | 9.69E-02 | NA | NA | NA | NA | NA |
| 14 | NA | NA | NA | NA | NA | NA | NA | NA | NA | NA |
| 15 | -0.16667 | 19 | 11 | 3.64E-02 | 4.39E-02 | NA | NA | NA | NA | NA |

**Supplementary table 1.** Clonal analysis of OHS1 and OHS2 mutations of IMGHV3-23*01. For the within-clone analysis, we proceeded as follows. Within each clone, we made pairwise comparisons between every sequence and created a list of ancestral-derived pairs for each pair where the mutations of one sequence (ancestral) were a subset of the other (assumes there are no reverse mutations), also noting the number of mutations from ancestral to derived. Clones were then categorized as belonging either to a case group, where “Site 1” (e.g. the G in AGCT) is mutated, and a control group where it is not mutated, ignoring clones where both types were found. We then compared the mean mutation frequency of “Site 2” (e.g. the adjacent C in AGCT) in case vs control, making the comparison for all pairs where the number of accumulated mutations match (Column “distance from the ancestral sequence”); also, only distances for which we had at least 10 clones in both case and corresponding control groups were considered. For each AGCT site, two conditions were considered: where the G in AGCT was “Site 1” and the neighboring C was “Site 2” (labeled as “OHS1/G-C” and “OHS2/G-C”), and vice versa (“OHS1/C-G”, “OHS2/C-G”). Furthermore, we only compared groups having the same mutational distance. Each table (for OHS1 and OHS2 respectively) shows the mean shift (difference case-control), number of clones in each group for that distance and the corresponding P-value (Mann-Whitney test) before and after FDR correction (BH method). Condition/distance pairs, where mutability shift was negative (as predicted), are highlighted in dark green if this shift was significant and in light green if it was not. Case/distance pairs, where mutability shift was positive, are highlighted in red.

For each of the four considered conditions we performed a Wilcoxon signed-rank test in order to check if the set of mean shifts for a given condition is significantly negative. It resulted in the following p-values: OHS1/G-C - 0.0017, OHS1/C-G - 0.125 (only 4 numbers in the set), OHS2/G-C - 0.0023, OHS2/C-G - 0.002.

| $1^{st}$ | $2^{nd}$ | Trimer | Polη overlap potential | | | GC |
| --- | --- | --- | --- | --- | --- | --- |
|  |  |  | Polη hotspots | ANC | Σ |  |
| W/S | R/Y | WRC | 3 | 1 | 4 | 1 |
|  |  | WYC | 1 | 1 | 2 | 0 |
|  |  | SRC | 0 | 0 | 0 | 1 |
|  |  | SYC | 0 | 0 | 0 | 0 |
|  | K/M | WKC | 1 | 1 | 2 | 1 |
|  |  | WMC | 3 | 1 | 4 | 0 |
|  |  | SKC | 0 | 0 | 0 | 1 |
|  |  | SMC | 0 | 0 | 0 | 0 |
|  | W/S | WWC | 4 | 1 | 5 | 0 |
|  |  | WSC | 0 | 1 | 1 | 1 |
|  |  | SWC | 0 | 0 | 0 | 0 |
|  |  | SSC | 0 | 0 | 0 | 1 |
| R/Y | R/Y | RRC | 1 | 1 | 2 | 1 |
|  |  | RYC | 0 | 1 | 1 | 0 |
|  |  | YRC | 2 | 0 | 2 | 1 |
|  |  | YYC | 1 | 0 | 1 | 0 |
|  | K/M | RKC | 0 | 1 | 1 | 1 |
|  |  | RMC | 1 | 1 | 2 | 0 |
|  |  | YKC | 1 | 0 | 1 | 1 |
|  |  | YMC | 2 | 0 | 2 | 0 |
|  | W/S | RWC | 1 | 1 | 2 | 0 |
|  |  | RSC | 0 | 1 | 1 | 1 |
|  |  | YWC | 2 | 0 | 2 | 0 |
|  |  | YSC | 0 | 0 | 0 | 1 |
|  |  |  |  |  |  |  |
|  |  |  |  |  |  |  |
| K/M | R/Y | KRC | 2 | 0 | 2 | 1 |
|  |  | KYC | 1 | 0 | 1 | 0 |
|  |  | MYC | 0 | 1 | 1 | 0 |
|  |  | MRC | 1 | 1 | 2 | 1 |
|  | K/M | KKC | 1 | 0 | 1 | 1 |
|  |  | KMC | 2 | 0 | 2 | 0 |
|  |  | MKC | 0 | 1 | 1 | 1 |
|  |  | MMC | 1 | 1 | 2 | 0 |
|  | W/S | KWC | 2 | 0 | 2 | 0 |
|  |  | KSC | 0 | 0 | 0 | 1 |
|  |  | MWC | 1 | 1 | 2 | 0 |
|  |  | MSC | 0 | 1 | 1 | 1 |

**Supplementary Table 2.** Enumeration of all possible 𝛼𝛽C trimers where 𝛼 and 𝛽 allow for two nucleotides each using IUPAC degenerate nucleotide scheme. The “Polη hotspots” column contains the number of possible Polη hotspots contained in a given motif. For example, the 4 possible WRC motifs contain 3 Polη hotspots in total: AAC contains one (AA), TAC contains 2 (TA and TA), TGC and AGC do not contain any. The “ANC” column shows if a given motif allows for an A in the first position, which might be a potential Polη hotspot, if the 5’ nucleotide to the motif is W. This is the only possibility for a potential Polη hotspots – for A in the second position we already know its 5’ nucleotide and for T in either first or second position we know its 3’ nucleotide, so if they are hotspots, they were already counted in the “Polη hotspots” column. The sum of these two columns can be considered as a measure of an overall potential to overlap with a Polη hotspot for a given trimer. All the trimers have a relatively low Polη overlap potential (0-2) except for 3: WRC (4), WMC(4), WWC(5). However, out of these three motifs only WRC allows for an NGC sequence leading to potential DSBs. This suggests that the WRC motif is optimal for Polη overlaps while allowing DSBs.

|  | **G** | **G** | **T** | **A** | **G** | **C** | **A** | **C** |
| --- | --- | --- | --- | --- | --- | --- | --- | --- |
| Sequential | **G** | **G** | T | W |  |  |  |  |
| One-sided |  | **G** | **T** | W |  |  |  |  |
| One-sided |  | **G** | T | **A** |  |  |  |  |
| Mutual |  |  | **T** | **A** |  |  |  |  |
| One-sided |  |  | W | **A** | R | **C** |  |  |
| Mutual |  |  |  | W | **G** | **C** | W |  |
| Sequential |  |  |  | W | R | **C** | **A** |  |
| Sequential |  |  |  | W | R | **C** | R | **C** |

**Supplementary Table 3.** GGTAGCAC motif with description of the overlapping hotspots it contains.
